# Cell Types of the Human Retina and Its Organoids at Single-Cell Resolution

**DOI:** 10.1016/j.cell.2020.08.013

**Published:** 2020-09-17

**Authors:** Cameron S. Cowan, Magdalena Renner, Martina De Gennaro, Brigitte Gross-Scherf, David Goldblum, Yanyan Hou, Martin Munz, Tiago M. Rodrigues, Jacek Krol, Tamas Szikra, Rachel Cuttat, Annick Waldt, Panagiotis Papasaikas, Roland Diggelmann, Claudia P. Patino-Alvarez, Patricia Galliker, Stefan E. Spirig, Dinko Pavlinic, Nadine Gerber-Hollbach, Sven Schuierer, Aldin Srdanovic, Marton Balogh, Riccardo Panero, Akos Kusnyerik, Arnold Szabo, Michael B. Stadler, Selim Orgül, Simone Picelli, Pascal W. Hasler, Andreas Hierlemann, Hendrik P.N. Scholl, Guglielmo Roma, Florian Nigsch, Botond Roska

**Affiliations:** 1 Institute of Molecular and Clinical Ophthalmology Basel, 4031 Basel, Switzerland; 2 Friedrich Miescher Institute for Biomedical Research, 4058 Basel, Switzerland; 3 Novartis Institutes for Biomedical Research, 4056 Basel, Switzerland; 4 Department of Ophthalmology, University of Basel, 4031 Basel, Switzerland; 5 Swiss Institute of Bioinformatics, 4058 Basel, Switzerland; 6 Bio Engineering Laboratory, Department of Biosystems Science and Engineering of ETH Zurich, 4058 Basel, Switzerland; 7 Department of Ophthalmology, Semmelweis University, 1085 Budapest, Hungary; 8 Department of Anatomy, Histology and Embryology, Semmelweis University, 1085 Budapest, Hungary; 9 Wilmer Eye Institute, Johns Hopkins University, Baltimore, MD 21287, USA

**Keywords:** organoid, retina, single cell sequencing, transcriptome, eye disease, retinal organoid, macular degeneration, organoid development, human retina, synaptic function

## Abstract

Human organoids recapitulating the cell-type diversity and function of their target organ are valuable for basic and translational research. We developed light-sensitive human retinal organoids with multiple nuclear and synaptic layers and functional synapses. We sequenced the RNA of 285,441 single cells from these organoids at seven developmental time points and from the periphery, fovea, pigment epithelium and choroid of light-responsive adult human retinas, and performed histochemistry. Cell types in organoids matured *in vitro* to a stable “developed” state at a rate similar to human retina development *in vivo*. Transcriptomes of organoid cell types converged toward the transcriptomes of adult peripheral retinal cell types. Expression of disease-associated genes was cell-type-specific in adult retina, and cell-type specificity was retained in organoids. We implicate unexpected cell types in diseases such as macular degeneration. This resource identifies cellular targets for studying disease mechanisms in organoids and for targeted repair in human retinas.

## Highlights

• Light-sensitive, multilayered human retinal organoids with functional synapses
• 285,441 transcriptomes from light-responsive human retinas and retinal organoids
• Organoid cell types converge to adult peripheral retinal cell types
• Linking retinal diseases to human retinal and retinal organoid cell types

## Graphical Abstract

**Figure:**
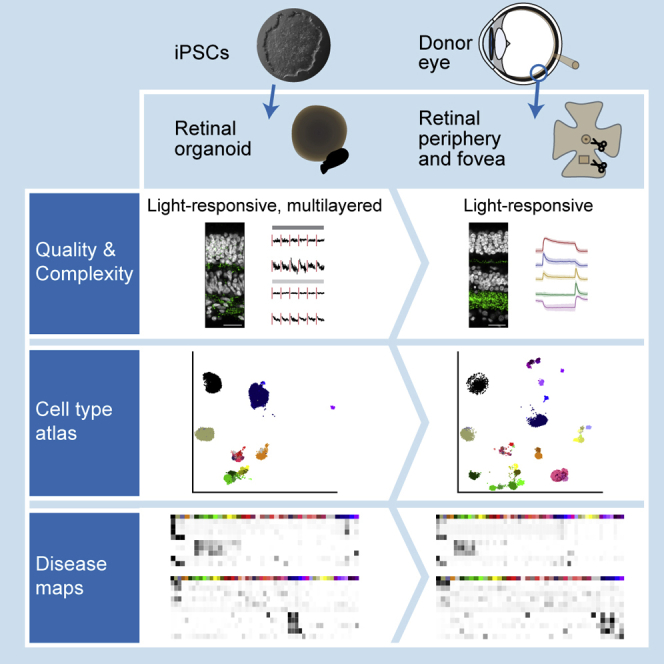

## Teaser

Light-sensitive, multilayered human retinal organoids with functional synapses are developed and benchmarked against human retinas obtained under conditions that preserve retinal integrity.

## Introduction

Human organoids are 3D cellular ensembles that are grown *in vitro* from adult or pluripotent stem cells and reproduce some morphological, functional, and transcriptomic features of human organs (Clevers, 2016; Lancaster and Knoblich, 2014a). Organoids engineered to harbor disease-causing mutations or grown directly from patient cells could provide mechanistic insights into diseases.

Human organs consist of many specialized cell types and a number of studies compared organoids to their target organ (Clevers, 2016; Lancaster and Huch, 2019). In the context of organ development, single-cell RNA sequencing has been employed to study how cell type differentiation in organoids compares to the developing target organ (Bhaduri et al., 2020; Brazovskaja et al., 2019; Camp et al., 2017; Lu et al., 2020; Sridhar et al., 2020; Tanaka et al., 2020). However, with few exceptions (Camp et al., 2017; Subramanian et al., 2019), it is not well understood how the transcriptomes of cell types in organoids converge toward the cell type transcriptomes of the adult organ. Nor is it well understood which disease genes retain their specificity for cell types between the target organ and its organoids or to what extent the function of cell types and their circuits are retained in organoids. How organoids are employed as a model system of diseases in adults will be guided by the answers to these questions.

The retina is a relevant model system to address these questions because its cell types have been extensively studied (Masland, 2012), and retinal organoids can be grown from human pluripotent stem cells (Meyer et al., 2011; Nakano et al., 2012; Zhong et al., 2014). Furthermore, many genes have been described that cause or contribute to vision-impairing monogenic and complex retinal diseases, such as retinitis pigmentosa and macular degeneration (Ferrari et al., 2011; Fritsche et al., 2016; Ran et al., 2014).

Retinas of humans have two distinct regions. The retinal periphery has low spatial acuity and is responsible for night-vision and different aspects of motion vision. The fovea (or macula) (Bringmann et al., 2018) is at the retinal center and drives high spatial acuity vision that is essential for reading and face recognition. Primates are the only mammals with a fovea. Retinal cells in both periphery and fovea can be divided into morphologically (Bae et al., 2018), functionally (Baden et al., 2016; Dacey et al., 2003; Roska and Werblin, 2001), and transcriptomically (Macosko et al., 2015; Peng et al., 2019; Shekhar et al., 2016; Siegert et al., 2012) different cell classes that are further divisible into cell types. The neural retina contains five layers (Dowling, 2012). Cell bodies are arranged in three distinct layers: photoreceptors in the outer nuclear layer, horizontal/bipolar/amacrine/Müller cells in the inner nuclear layer, and amacrine/ganglion cells in the ganglion cell layer. Retinal neurons make synaptic connections in two interjacent layers: the outer and inner plexiform layers. Embedded in this layered structure are astrocytes, microglial cells, and the retinal vasculature, which is composed of endothelial cells and pericytes. Conserved across vertebrates, the five-layered neural retina is covered on the photoreceptor side by the retinal pigment epithelium and by the choroid, which contains endothelial cells, pericytes, fibroblasts, and melanocytes (Nickla and Wallman, 2010). We refer to the neural retina, the retinal pigment epithelium and the choroid together as “the retina.”

Cells in the retina display a number of functionally important subcellular specializations. In photoreceptors, for example, the outer segment captures light, the connecting cilium enables the transport of outer-segment specific molecules, the inner segment hosts mitochondria that produce energy, and the ribbon synapse permits graded signal transmission of sensory information. The distinguishing characteristics of the cell types and their subcellular specializations are associated with the expression of specific genes that are frequently implicated in retinal disease (Ran et al., 2014). The transcriptomes of some adult human retinal cell types have been described (Liang et al., 2019; Lukowski et al., 2019; Menon et al., 2019; Voigt et al., 2019); however, the cells were sampled from donor retinas post mortem after several hours in an ischemic state. Ischemia leads to irreversible damage to the retina within 20 min (Osborne et al., 2004).

Human retinal organoids have been used to study retinal development and disease (Foltz and Clegg, 2019; Kruczek and Swaroop, 2020) but, unlike the real human retina, currently used organoids are not five-layered and have not been shown capable of rapidly transmitting light responses synaptically to inner retinal layers. Additional barriers to modeling genetic diseases of the retina are the difficulty of producing organoids in large quantities and the lack of a comprehensive quantitative comparison of gene expression between cell types in organoids and adult human retinas (Collin et al., 2019; Kim et al., 2019; Lu et al., 2020). For cell types in retinal organoids, it is therefore not well understood whether and when their gene expression matches that of the adult human retina. For many retinal disease genes, it is not known in which cell types of the adult human retina they are expressed or at what age organoid cell types reproduce this expression.

Here, we report the development of retinal organoids with three nuclear and two synaptic layers from human induced pluripotent stem cells (iPSCs) and their production in large quantities. The photoreceptors of the organoids responded to light and transmitted visual information synaptically, generating light responses in second or third order retinal cells. We obtained single-cell transcriptomes from 110,862 cells dissociated from developing human multilayered organoids at seven different time points spanning the 38 weeks of human gestation and up to week 46. Analysis of these transcriptomes revealed progressive maturation of retinal cell classes and showed that organoid transcriptomes reached a stable, “developed” state between weeks 30 and 38. The rate of transcriptomic changes during organoid development was similar to the developing human retina *in vivo*. We show that adult human retinal cell type transcriptomes change rapidly post mortem in ischemia, and we developed a procedure to obtain adult human retinas that were exposed to less than 5 min of ischemia and maintained light responses and functional retinal circuits for 16 h *ex vivo*. We sequenced RNA from 174,579 single cells from the peripheral and foveal retina, including the pigment epithelium and choroid. Comparing periphery to fovea, we identified regional characteristics of cell types and, by comparing organoid to organ, we showed that transcriptomes of organoid cell types converge to those of adult human peripheral retinas. In the context of cell types, we also compared developed organoids and adult human retinas in their expression of genes associated with retinal diseases. The resulting genetic disease maps showed retinal diseases to be cell-type-specific and that cell-type specificity is preserved in organoids. The resources we describe here provide iPSC lines and a high-throughput method to build retinal organoids, with reproducible organ features and transcriptomes, for modeling retinal disease. Furthermore, we provide a comparative atlas of cell-type transcriptomes of human retinal organoid and healthy adult human retina that allows identification of cellular targets for studying disease mechanisms in organoids and targeted repair in adult human retinas.

## Results

### Multilayered Human Retinal Organoids in Quantity

**Figure 1:**
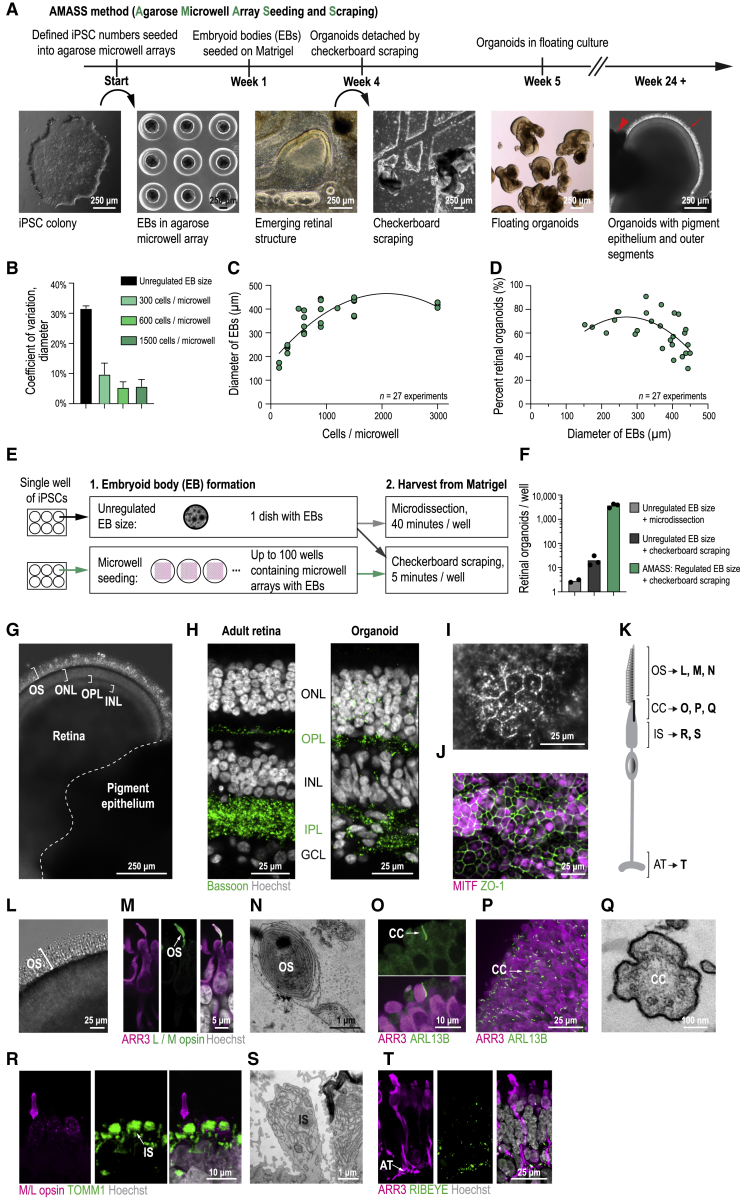
Multilayered Human Retinal Organoids Produced in Quantity (A) Timeline of the AMASS organoid protocol with example bright-field images at important stages. Red arrowhead, pigment epithelium; red arrow, outer segments. EB, embryoid body. (B) Variability of embryoid body diameter with different generation methods. Bars, average coefficient of variation. Error bars, SD. (C) Embryoid body diameter (day 7) versus the number of cells seeded per microwell. Points, average diameter of embryoid bodies (n = 12) within an independent experiment. Line, quadratic fit. (D) Percentage of all organoids that were retinal organoids (week 6) versus embryoid body diameter (day 7). Points, experiments. Line, quadratic fit. (E) Schematic visualizing how AMASS improves yield from iPSCs using microwell array seeding and checkerboard scraping. (F) Comparison of organoid yield per well of iPSCs using different methods, weeks 20–38. Points, experiments. (G) Bright-field image of an organoid. OS, outer segment; ONL, outer nuclear layer; OPL, outer plexiform layer; INL, inner nuclear layer. (H) Confocal images. Left: adult retina. Right: organoid. Green, antibody against Bassoon (synaptic marker); white, Hoechst (nucleus marker). IPL, inner plexiform layer; GCL, ganglion cell layer. (I) Bright-field image of organoid pigment epithelial cells with black pigmentation. (J) Confocal image of pigment epithelial cells (maximum intensity projection). Magenta, MITF antibody; green, ZO-1 antibody (pigment epithelial cell markers). (K) Illustration of photoreceptor subcellular compartments. OS, outer segment; CC, connecting cilium; IS, inner segment; AT, axon terminal. (L–T) Organoid photoreceptors. (L–N) Outer segment. (L) Bright-field image. (M) Confocal images. Magenta, ARR3 antibody (cone marker); green, L/M opsin antibody (cone outer segment marker); white, Hoechst (nucleus marker). (N) Electron microscope image. Diagonal section of membrane discs. (O–Q) Connecting cilium. (O and P) Confocal images. Magenta, ARR3 antibody; green, ARL13B antibody (cilium marker). (P) Maximum intensity projection showing organoid surface. (Q) Electron microscope image. (R and S) Inner segment. (R) Confocal image. Magenta, L/M opsin antibody; green, TOMM1 antibody (mitochondrion marker). (S) Electron microscope image. (T) Axon terminal. Confocal image. Magenta, ARR3 antibody; green, RIBEYE antibody (ribbon synapse marker). All data from F49B7 organoids. See also Figure S1, Table S1, and Video S1.

**Figure S1:**
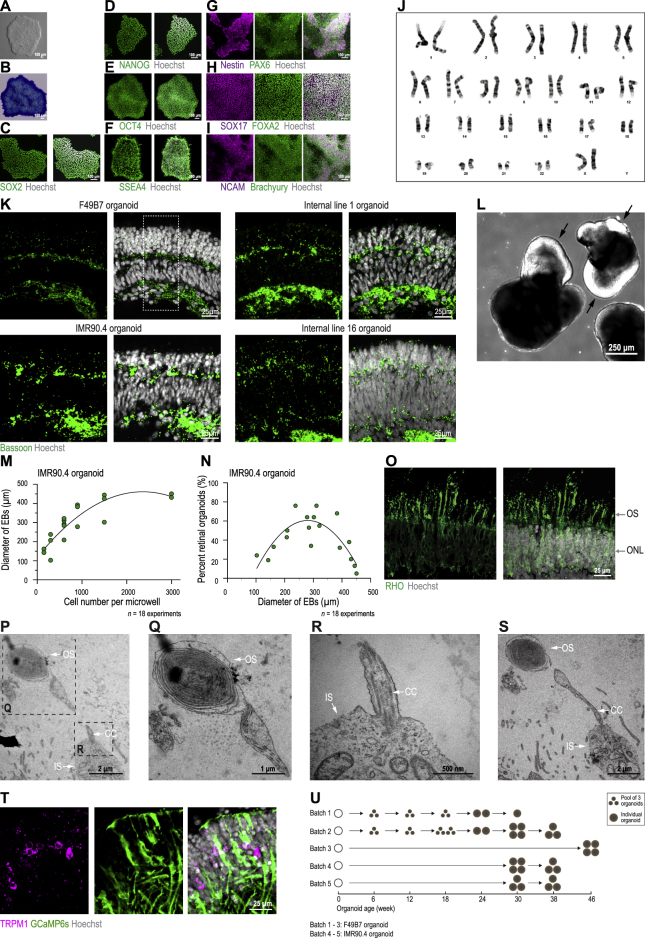
Characterization of F49B7 iPSCs and Retinal Organoids from Several iPSC Lines, Related to Figures 1, 2, and 3 and Table S1 (A) Bright-field image of F49B7 iPSC colony. (B) Bright-field image of iPSC colony. Blue stain, alkaline phosphatase (pluripotency marker). (C – F) Confocal images of iPSCs. Green, antibody for pluripotency markers; white, Hoechst (nucleus marker). (C) SOX2, (D) NANOG, (E) OCT4 and (F) SSEA4. (G – I) Confocal images of iPSCs directly differentiated into the three germ layers. White, Hoechst. (G) Ectoderm; magenta, Nestin; green, PAX6 (ectoderm markers). (H) Endoderm; magenta, SOX17; green, FOXA2 (endoderm markers). (I) Mesoderm; magenta, NCAM; green, Brachyury (mesoderm markers). (J) G-banded karyotyping of F49B7 iPSCs. (K) Different iPSC lines generating 5-layered retinal organoids. Confocal images. Green, Bassoon antibody (synaptic marker); white, Hoechst (nucleus marker). Boxed area, outline of F49B7 image shown cropped in Figure 1. (L) Retinal neuroepithelium (arrows) on a five-week old organoid. (M) Embryoid body diameter (day 7) versus the number of cells seeded per microwell. Points, mean diameter of embryoid bodies (n = 12) within an independent experiment. Line, quadratic fit. (N) Percentage of all organoids that were retinal organoids (week 6) versus embryoid body diameter (day 7). Points, experiments. Line, quadratic fit. (O) Confocal image. Green, RHO antibody (rod outer segment marker); white, Hoechst (nucleus marker). OS, outer segment; ONL, outer nuclear layer. (P – S) Electron microscope images of photoreceptor outer segment (OS; P, Q, S), inner segment (IS; P, R, S) and connecting cilium (CC; P, R, S). P and S are from serial sections. (T) Confocal image. Magenta, TRPM1 antibody (ON bipolar cell marker); green, GFP antibody (GCaMP6s); white, Hoechst (nucleus marker). (U) Scheme of the time course with which organoids were sampled for single-cell RNA sequencing. Data in A – J, K, L, O, P – S and T are from F49B7 organoids, data in K, M and N are from IMR90.4 organoids.

We aimed to develop human retinal organoids with three nuclear and two synaptic layers from iPSCs, building on the observation that iPSC lines vary in their potential to produce retinal organoids: some gave rise to organoids while others did not. As organoid differentiation is a nonlinear process (Dahl-Jensen and Grapin-Botton, 2017), the qualitatively different outcomes could be due to differences in the initial condition of the iPSCs, namely their genetic origin or their epigenetic and transcriptomic states at the time of differentiation (Kilpinen et al., 2017; Ortmann and Vallier, 2017). Therefore, we screened 23 iPSC lines for the formation of multilayered retinal organoids. Eight of 23 lines formed organoids with a layered appearance under the light microscope that persisted for more than 100 days in culture (Figure 1; Table S1). In organoids from four lines, we detected the formation of three nuclear layers and two synaptic layers matching the cellular organization of the adult retina (Figures 1 and S1). This study focuses on the 01F49i-N-B7 (short name: F49B7) iPSC line (Figure S1), but we repeat some experiments with the IMR90.4 iPSC line.

Generation of embryoid bodies is the first step of organoid production (Figure 1A). However, embryoid bodies produced from iPSC aggregates vary in size (96 μm to 640 μm). To control the size of embryoid bodies, we generated them in agarose microwell arrays from a defined number of dissociated iPSCs, which reduced variability by a factor of 4.7 (Figure 1B) and allowed the average embryoid body size to be controlled by changing the number of cells seeded per microwell (Figures 1C, F49B7, and S1, IMR90.4). Embryoid body size strongly influenced the efficiency of retinal organoid production (Figures 1D, F49B7, and S1, IMR90.4).

After embryoid bodies have been seeded onto Matrigel and developed in 2D culture into optic-cup-like structures, a widely used approach to free them from Matrigel is manual microdissection (Zhong et al., 2014). This step is time-consuming, precluding the fabrication of organoids in large quantities. We evaluated whether dislodging the contents of the entire plate by scraping along a checkerboard pattern (Figure 1; Video S1) could improve throughput. Checkerboard scraping was faster than manual microdissection (5 min versus 40 min) and yielded significantly more organoids per plate (p = 2 × 10^−16^, exact rate ratio test, organoids from n = 17 wells of iPSCs) (Figures 1E and 1F). By combining microwell array seeding with checkerboard scraping, 3,700 ± 680 (mean ± SD, n = 3 experiments) retinal organoids can be generated from the iPSCs in a single well of a 6-well plate (Figure 1F). We name this method AMASS (agarose microwell array seeding and scraping).

Video S1. Demonstration of Organoid Checkerboard Scraping Method, Related to Figures 1 and S1

Retinal organoids contained patches of pigment epithelium (Figures 1I and 1J) and their photoreceptors displayed characteristic subcellular compartments (Figures 1K–1T and S1).

### Organoids Are Light Responsive and Contain Functional Synapses

**Figure 2:**
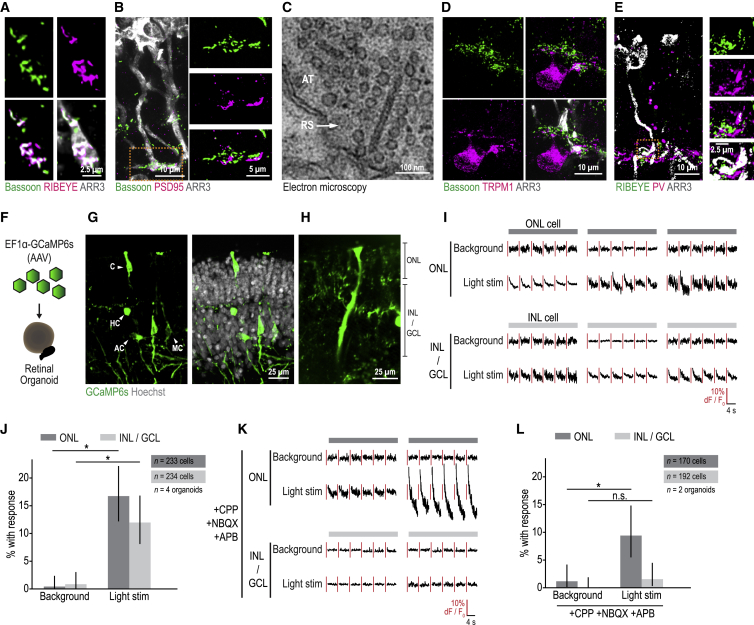
Organoids Are Light-Responsive and Contain Functional Synapses (A–E) Synapses in organoids. (A, B, D, and E) Cone axon terminals. Confocal images. White, ARR3 (cone marker). Green, antibody against Bassoon (ribbon synapse marker); RIBEYE (ribbon synapse marker). Magenta, antibody against RIBEYE; PSD95 (postsynaptic marker); TRPM1 (ON bipolar cell marker); PV (horizontal cell marker). (C) Electron microscope image. AT, photoreceptor axon terminal; RS, ribbon synapse. (F) Schematic of organoid infection with adeno-associated viral vectors (AAV) expressing the calcium sensor GCaMP6s under the promotor EF1α. (G and H) Organoids expressing GCaMP6s. (G) Confocal image. Green, antibody against GFP detecting GCaMP6s. White, Hoechst (nucleus marker). C, cell with cone morphology; HC, cell with horizontal cell morphology; AC, cell with amacrine cell morphology; MC, Müller cell confirmed by counter-stain. (H) Two-photon microscope image. Optical cross-section of living organoid. Green, GCaMP6s. ONL, outer nuclear layer; INL, inner nuclear layer; GCL, ganglion cell layer. (I) Organoid calcium activity during background illumination and light stimulation. Top, cells from ONL. Bottom, cells from INL/GCL. Black lines, peri-stimulus calcium activity (dF/F_0_); line segments, individual trials. Red lines, scale bars. (J) Quantification of light responsive cells in ONL and INL/GCL. Error bars, 95% binomial confidence interval. (K and L) Organoid calcium activity during pharmacological block of glutamatergic synaptic transmission. CPP, (3-[(R)-2-carboxypiperazin-4-yl]-propyl-1-phosphonic acid); NBQX (2,3-dioxo-6-nitro-7-sulfamoyl-benzo[f]quinoxaline); APB, (2-amino-4-phosphonobutyric acid). (K) Responses from individual cells, as in (I). (L) Quantification of light responsive cells in ONL and INL/GCL, as in (J). All significance values from χ^2^ test. All data from F49B7 organoids. See also Figure S1.

To understand if organoid photoreceptors form synapses, we performed immunostainings for synaptic proteins. The ribbon synapse proteins Bassoon and RIBEYE co-localized at photoreceptor axonal terminals (Figure 2A) and the postsynaptic protein PSD95 was observed juxtaposed with Bassoon (Figure 2B). Electron microscopy of organoids showed ribbon synapses with vesicles docked (Figure 2C). We found cone photoreceptor axon terminals with ribbon synapse staining in close contact with bipolar cells (Figure 2D) and horizontal cells (Figure 2E). These results suggest the presence of synapses between photoreceptors and second-order retinal cells.

We assessed whether organoids contain synapses capable of transmitting light responses from photoreceptors to second or third order organoid cells. We expressed the calcium sensor GCaMP6s under the control of the EF1α promoter in cells across all retinal layers using adeno-associated viruses (Figures 2F and 2G). We performed live two-photon laser imaging of organoids, visualizing an optical cross-section that encompassed the outer nuclear, inner nuclear and ganglion cell layers (Figure 2H). We stimulated photoreceptors with the two-photon laser used for imaging, which was shown to evoke light responses (Baden et al., 2013, 2016; Hsiang et al., 2017; Ruminski et al., 2019). As a control, background activity was measured by continuous two-photon imaging after photoreceptors adapted to the stimulation.

In the outer nuclear layer, we observed repeatable responses to light in 16.7% of detected cells, significantly above background activity (p = 2.6 × 10^−11^, χ^2^ test, n = 233 cells, n = 4 organoids) (Figures 2I and 2J). The fluorescence of the recorded cells decreased during stimulation and blocking glutamatergic synaptic transmission did not abolish light responses (Figures 2K and 2L). These results suggest that the recorded organoid cells in the outer nuclear layer are light sensitive photoreceptors, which hyperpolarize in responses to light.

Within the inner nuclear and ganglion cell layers, 12.0% of cells (“inner organoid cells”) repeatedly responded to light, significantly above background activity (p = 1.6 × 10^−7^, χ^2^ test, n = 234 cells, n = 4 organoids) (Figures 2I–2J). The fluorescence of inner organoid cells decreased during light stimulation and blocking glutamatergic synaptic transmission abolished the light responses to the level that was observed at background (1.6% of cells, p = 0.081, χ^2^ test, n = 192 cells, n = 2 organoids) (Figures 2K and 2L). Therefore, inner organoid cells are synaptically driven by the organoids’ photoreceptors and the recorded inner organoid cells are all “OFF” cells. ON bipolar cells were not well-represented in our recordings, with GCaMP6s observed in only 0.9% of organoid ON bipolar cells (Figure S1, n = 116 cells, n = 5 organoids), the same lack of ON bipolar cell targeting with EF1α was observed in the mouse retina (Cronin et al., 2014).

### Organoids Stabilize to a Developed State

**Figure 3:**
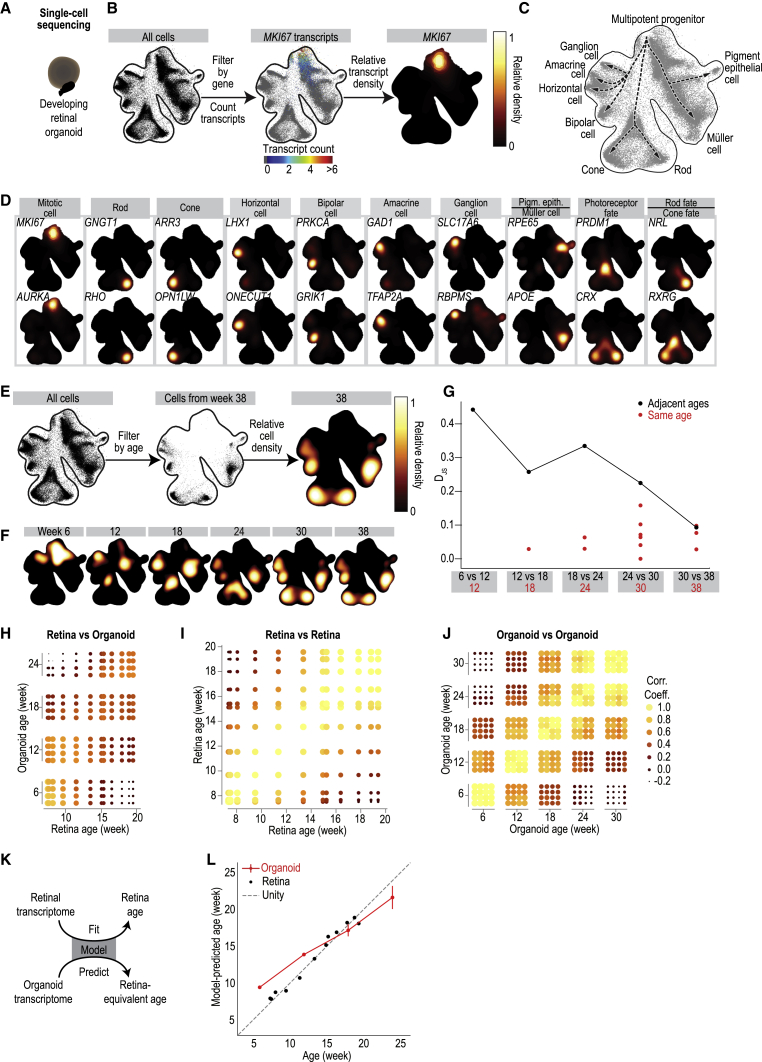
Organoid Transcriptome Stabilizes *In Vitro* with a Development Rate that Matches that of the Retina *In Vivo* (A) Retinal organoid single-cell sequencing. (B) Left: scVis map of cells across weeks 6–38. Each point represents the transcriptome of a single cell. Black line, isodensity contour. Middle: scVis map with transcript counts of a single gene (here, mitosis marker *MKI67*) color-coded; colormap at bottom. Right: heatmap of relative transcript density for *MKI67*; colormap at right. (C) Cell classes marked on scVis map according to (D). Arrows, inferred developmental trajectories. (D) Heatmap of relative transcript density for genes marking mitotic cells, retinal cell classes, and retinal precursors. (E) Left: scVis map of cells across all ages. Middle: a black point represents a cell from an organoid of a specific age (here week 38). Right: heatmap of relative cell density at week 38; colormap at right. (F) Heatmap of relative cell density at six different ages. (G) Black line, Jensen-Shannon divergence (*D*_JS_) of organoid transcriptomes at adjacent ages. Red dots, comparison of individual organoids from the same age and batch. (H–J) Correlation in gene expression at different ages. Each point is a correlation coefficient between two samples. Point size and color, correlation strength; scale at right. (H) Developing organoid versus developing retina. (I) Retina versus retina. (J) Organoid versus organoid. (K) A model, trained to predict retina age based on retinal transcriptome data, is applied to predict retina-equivalent age of organoid samples. (L) Red line, model-predicted retina-equivalent age of organoids versus their age in culture (mean ± 3 SEM). Black points, model-predicted age of retinas versus their developmental age. Dashed gray line, one-to-one correspondence between age and model-predicted age. All organoid data from F49B7 organoids. See also Figures S1 and S2.

We approached the question of when organoids in culture are “developed” by performing single-cell RNA sequencing at six time points during organoid development: 6, 12, 18, 24, 30, and 38 weeks (Figure S1). We analyzed 62,136 cells from F49B7 organoids using the 10x Genomics Chromium platform (n = 12 individual organoids, n = 7 pooled organoids). We embedded the transcriptomes of organoid cells, pooled across all time points, within a 2D map using the deep learning-based scVis algorithm (Ding et al., 2018) (Figure 3B). Each point on the map represents the transcriptome of a single cell, and adjacent points correspond to transcriptomes that are similar to each other.

**Figure S2:**
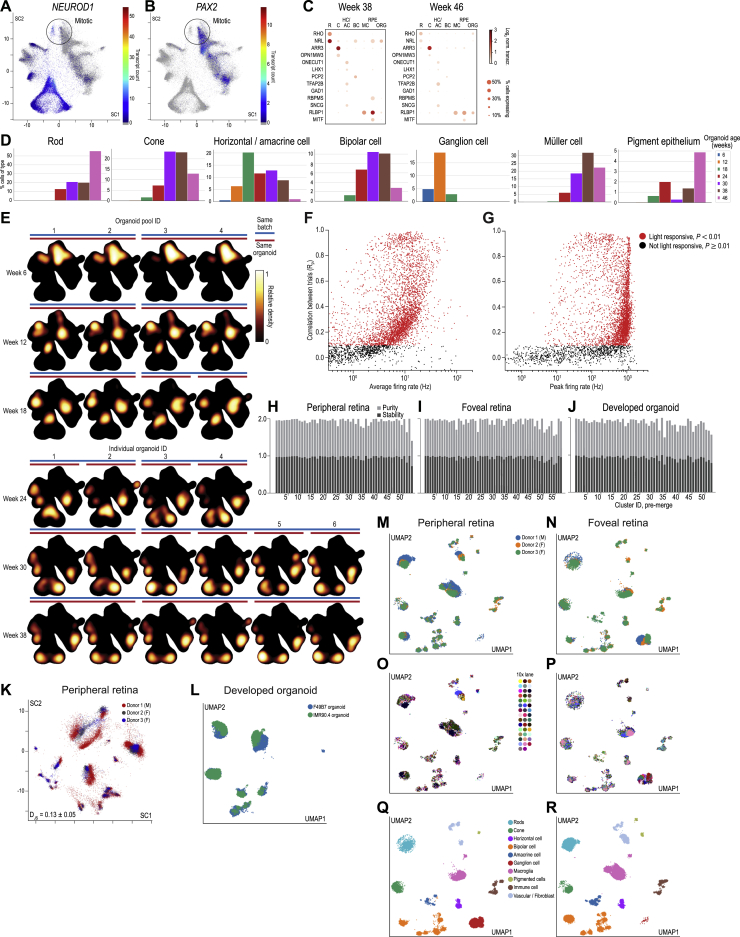
Reproducibility of Organoids, Firing Rate Statistics of Adult Retinal Ganglion Cells, Infomap Clustering Quality, and Sources of Variability in Adult Retinal UMAP Maps, Related to Figures 3, 4, and 5 Location of transcripts of (A) neurogenic and neuronal marker *NEUROD1* (60 × upregulated, p = 1.8 × 10^−308^) and (B) gliogenic marker *PAX2* (37 × upregulated, p = 5.8 × 10^−12^) in two pools of mitotic progenitor cells on organoid scVis map. Circles, cells expressing mitotic markers; color, transcript count; colormap at right. (C) Expression of cell type markers in week 38 (left) and week 46 (right) organoids. Colormap and legend at right. (D) Cell type composition in organoids at week 46 compared to other ages. Legend at right. (E) Heatmap of relative cell density in 28 different samples with indicators for organoid age (row), pooling method (top, pool; bottom, individual), batch (blue bar), and replicates from the same organoid (red bar). (F, G) Scatterplots comparing the correlation in ganglion cell firing rates across stimulus repetitions to their (F) average firing rate and (G) peak firing rate. Red, cells significantly light responsive (p < 0.01). Black, cells not light responsive (p ≥ 0.01). (H – J) Cluster quality was evaluated in a bootstrap process to evaluate the Infomap clusters of (H) peripheral retina, (I) foveal retina and (J) developed organoids. Light gray bars, cluster purity. Dark gray bars, cluster stability. (K) Peripheral retinal cell transcriptomes in scVis map, variation across donors. Points, single cell transcriptomes. Color, donor. (L) UMAP map of developed organoids labeled according to cell line. (M – R) The peripheral (left) and foveal (right) UMAP maps labeled according to (M – N) donor identity and sex; M, male; F, female, (O – P) 10x Chromium lane and (Q – R) cell class annotation used during subclustering.

Within the map, single-cell transcriptomes were distributed along a leaf-like manifold. We located on this manifold the transcriptomes of cells expressing known genetic markers of retinal progenitors or specific retinal cell classes (Figures 3B–3D and S2). An ordered series of cell transcriptomes across the leaf-like manifold captured a progression of gene expression patterns reached by cells as they develop from multipotent progenitors to differentiated retinal cell classes (Figure 3C).

We next investigated the transcriptomes of cells at different ages within the manifold and how their locations change with time (Figures 3E and 3F). The 6-week-old cell transcriptomes were concentrated in the manifold’s upper region, and at each subsequent time point, the distribution shifted closer to the regions containing transcriptomes of differentiated cell classes. The temporal order of appearance of neural retinal cells was ganglion cells, photoreceptor precursors, horizontal cells, amacrine cells, bipolar cells, and Müller cells. Although ganglion cells were abundant at 12–18 weeks, they were rare by week 24: ganglion cell marker *SLC17A6* was detected in 0.36% of organoid cells between weeks 24 and 38. To quantify changes in gene expression within the 6- to 38-week time window of organoid development, we calculated the Jensen-Shannon divergence (*D*_JS_) of distributions shown on scVis maps between organoids at the same or different ages (Figure S2). *D*_JS_ provides a measure of dissimilarity between distributions, which takes a value of zero for perfectly overlapping distributions and one for non-overlapping distributions. The mean *D*_JS_ between individual organoids from the same batch and age stayed below 0.1 through week 30 (*D*_JS_ = 0.073 ± 0.040, mean ± SD) and 38 (*D*_JS_ = 0.068 ± 0.030) (Figure 3G) that is similar in magnitude to the *D*_JS_ between retinas from individual human donors (*D*_JS_ = 0.129 ± 0.045, Figure S2). The curve of *D*_JS_ plotted between adjacent time points as a function of age trended downward with increasing time in culture (Figure 3G), reaching its minimum (*D*_JS_ = 0.093) between weeks 30 and 38, a value that was within one standard deviation of the *D*_JS_ values for transcriptomes observed at the same age and between individual donors.

We further evaluated transcriptome stabilization with week 46 organoids (n = 20,802 cells, n = 3 individual organoids). Week 46 organoids had fewer bipolar cells, horizontal/amacrine cells, and cones compared to week 38, but more rods (Figure S2). Week 46 organoids were composed of 1.1% inhibitory interneurons, a large decrease from 8.9% in week 38 organoids. In addition, the expression of cell type markers was reduced from week 38 to 46, including the expression of rod markers *RHO* (week 38, 57.4% of rods expressing; week 46, 42.1%; p = 6 × 10^−33^, Mann-Whitney U of normalized transcript counts) and *NRL* (week 38, 98.4%; week 46, 38.3%; p = 2 × 10^−308^) (Figure S2).

Thus, the gene expression patterns of organoids reproducibly stabilized between week 30 and 38 in a state containing most retinal cell classes. Additional time in culture did not further develop organoids, instead appearing to decrease cell type diversity and expression of cell type markers. On this basis, we refer to organoids at the 30- and 38-week time points as “developed organoids.”

### Matching Rates of Organoid and Retina Development

The rate of development of an organoid *in vitro* may differ from that of the retina *in vivo*. To compare the two rates, we first cross-correlated the bulk transcriptomes of organoids, obtained by combining single-cell transcriptomes, to published bulk transcriptomes of human retina between 7 and 20 weeks in developmental age (Hoshino et al., 2017) (Figures 3H–3J). We created a linear model that was trained to predict retinal age using the retinal transcriptomes (Figure 3K). The model was validated on the transcriptomes of the developing retina (Figure 3L). The trained model was then applied to the organoid transcriptomes to predict their retina-equivalent developmental age. Remarkably, when the model-predicted, retina-equivalent developmental ages of the organoids were plotted against their actual developmental ages, the data points were close to the unity line (Figure 3L). Thus, the rates of development of organoids *in vitro* and retina *in vivo* are similar.

### Cell-Type Transcriptomes in Functional Adult Human Retinas and Developed Organoids

**Figure 4:**
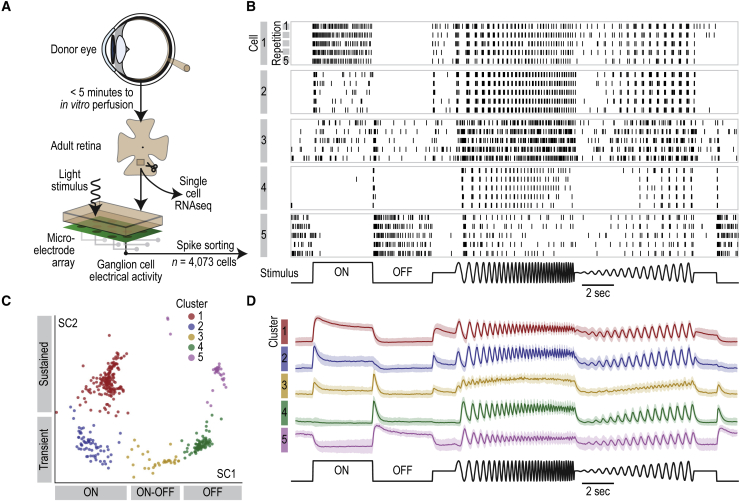
Post Mortem Adult Retina with Light Responses (A) Schematic of the procedure to obtain adult retinas for single-cell RNA sequencing and electrophysiology. (B) Vertical lines, action potentials fired by five example ganglion cells (horizontal boxes) during five repetitions (rows within boxes) of a light stimulus (at bottom). (C) scVis map of light responses from a sample of ganglion cells at the same retinal location; each point represents the firing rate characteristics of an individual cell in response to visual stimulation. Colors, Infomap clusters. Labels at the two axes, response characteristics of cells in that region of the scVis map. (D) Colored lines, normalized firing rate of cells within each cluster during the stimulus. Shaded regions, ±1 SD. Colors, Infomap clusters as in (C). See also Figure S2.

Organoids have distinct and transcriptomically defined cell classes by week 30. To determine how close the transcriptomes of these cell classes are to those of the adult retina, we sequenced RNA of single cells from light-sensitive retinas of human adult multi-organ donors. A post mortem retina is irreversibly damaged after 20 min of ischemia (Osborne et al., 2004), therefore we combined rapid surgery and eye-cup preparation with an oxygenation and perfusion-providing transportation device to reduce the ischemic window experienced by donor retinas to less than 5 min (Figure 4A). We tested the functional state of the peripheral neural retina and, in parallel, used neural retina, retinal pigment epithelium, and choroid from the periphery and fovea for single-cell RNA sequencing.

We assessed the state of the peripheral neural retina by quantifying different aspects of light-induced activity, using a high-density microelectrode array to record ganglion cells that fired action potentials during visual stimulation. Remarkably, 82% of the recorded ganglion cells showed significant light responses (3,550 cells of n = 4,073; p < 0.01, Pearson correlation) to a series of spatially uniform steps, frequency chirps, and intensity sweeps (Figure 4B). Of the responding cells, 50% had a peak firing rate above 775 Hz and 95% above 52 Hz (Figure S2). We observed five different ganglion cell response types including ON/OFF and transient/sustained (Figures 4C and 4D), consistent with an adult retina that is healthy at multiple levels in its circuitry (Field and Chichilnisky, 2007): the ON and OFF responses show that the split of information from photoreceptors to ON and OFF bipolar cells is intact, transient responses show functional feedback and feedforward inhibition from horizontal and amacrine cells, and the high firing rates show efficient light capture and transmission of information across retinal synapses.

**Figure 5:**
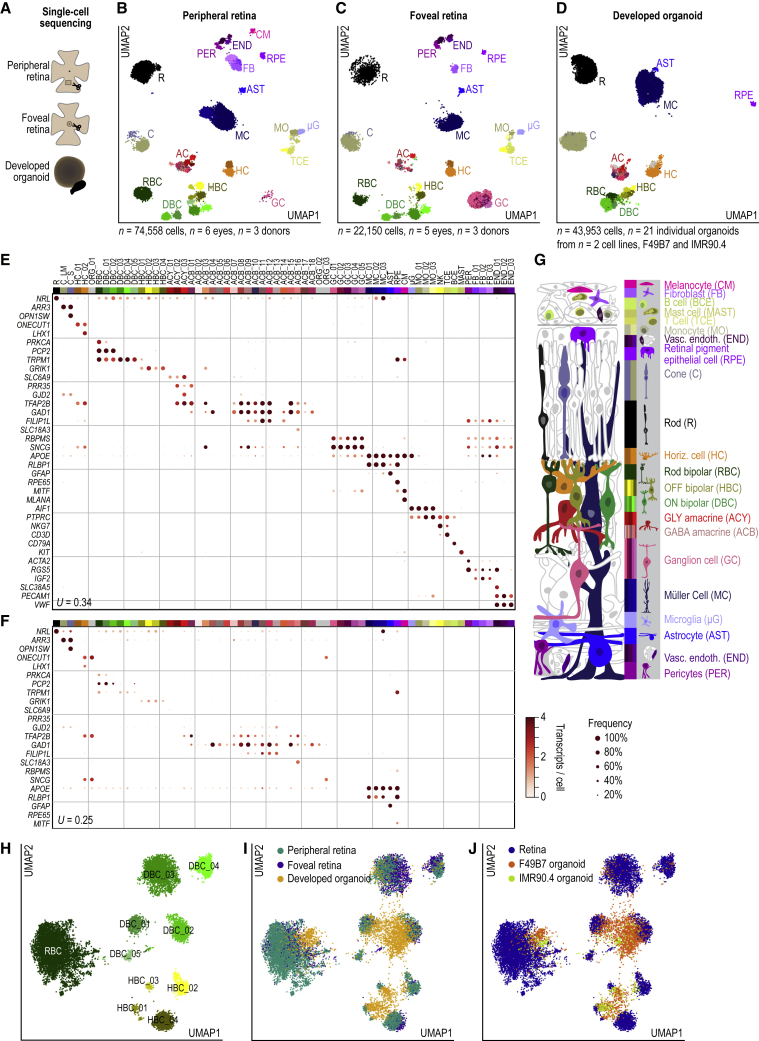
Cell Types of the Adult Human Retina and Its Organoids (A) Single cells sequenced from peripheral retina, foveal retina, and developed retinal organoids (weeks 30 and 38). (B–D) UMAP maps of transcriptomic cell types within (B) peripheral retina, (C) foveal retina, and (D) developed organoids. Points, transcriptome of single cell. Abbreviations/colors, cell classes, and types as in (G). (E and F) Expression of known marker genes (rows) within Infomap clusters (columns) for (E) adult retina, both peripheral and foveal and (F) developed organoids. Dot size, percent of cells expressing. Dot color, mean transcripts per cell. Legend and colormap at bottom right of F. *U*, uncertainty coefficient. (G) Illustration, retinal cell classes, and types. Color bar, cell types; shades of the same color, cell types within the same cell class. (H–J) Subclustering of bipolar cells in peripheral retina, foveal retina, and retinal organoid. Points, single bipolar cells. (H) Color, bipolar cell type. Peripheral and foveal retina. (I) Color, region of origin. (J) Color, tissue of origin. All organoid data from F49B7 and IMR90.4 organoids. See also Figures S2, S3, S4, S5, and S6 and Table S2.

From these light responsive adult retinas, we compared the transcriptomes of individual peripheral and foveal cells (total of 96,708 cells; n = 74,558 peripheral retinal cells from n = 6 eyes; n = 22,150 foveal retinal cells from n = 5 eyes; from 3 donors) with those of cells from developed organoids (n = 53,067 cells from n = 8 individual F49B7 and n = 13 individual IMR90.4 organoids). The average number of unique transcripts per cell was 2,508 in the peripheral retina, 7,146 in the foveal retina, and 4,096 in developed organoids. Sequencing results, count tables, and atlases are available in the Key Resources Table, Table S2, and at https://data.iob.ch. We identified transcriptome-based cell types by Infomap clustering of single-cell transcriptomes in the peripheral retina, foveal retina, and developed organoids. Clustering was performed hierarchically, first grouping related cell types and then clustering within each group to identify cell types. We quantified the quality of clustering by cluster purity and by cluster stability (Figure S2) (Shekhar et al., 2016). To visualize groups of transcriptomically similar cells, we performed UMAP embedding and represented Infomap clusters on the resulting map by different colors (Figures 5B and 5D).

**Figure S3:**
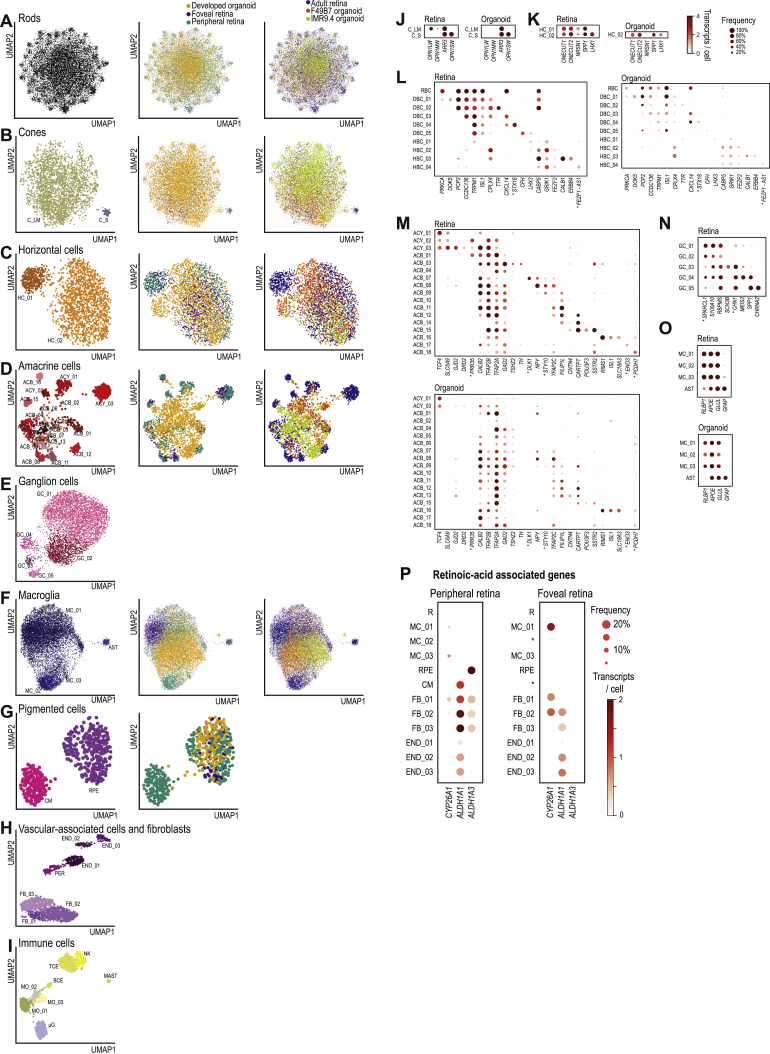
Identification of Retinal Cell Types, Related to Figure 5 and Table S2 (A – I) UMAP map of cell types, colored according to cell type (left), region of origin (middle), and tissue of origin (right). (A) Rods. (B) Cones. (C) Horizontal cells. (D) Amacrine cells. (E) Ganglion cells. (F) Macroglia. (G) Pigmented cells. (H) Vascular-associated cells and fibroblasts. (I) Immune cells. (J – O) Expression of known cell type marker genes (columns) by Infomap cell types (rows). (J) Cones. (K) Horizontal Cells. (L) Bipolar cells. (M) Amacrine cells. (N) Ganglion cells. (O) Macroglia. Dot size, percent of cells expressing. Dot color, mean transcripts per cell. Colormap and legend, right of panel K. Cell type acronyms as in Figure 5. ^∗^, cell-type markers not previously known. (P) Expression of retinoic acid pathway genes, implicated in patterning the fovea (da Silva and Cepko, 2017). Genes for retinoic acid synthesis (*ALDH1A1*, *ALDH1A3*) and catabolism (*CYP26A1*), expression levels in selected cell types. *CYP26A1* was expressed in foveal Müller cells and was significantly upregulated relative to the periphery (Mann-Whitney U test, *CYP26A1*: 6.3 × upregulated, p = 6 × 10^−176^). *ALDH1A3* alone was expressed in pigment epithelial cells, where it was highly upregulated in the periphery relative to the fovea (630 × upregulated, p = 2.5 × 10^−29^). Dot size, percent of cells expressing. Dot color, mean transcripts per cell. Right, colormap and legend. Cell type acronyms are from Figures 5B–5G. ^∗^, cell type not present in tissue.

We detected 65 transcriptome-based cell types (Infomap clusters), 53 in the peripheral retina (Figure 5B), and 41 in the foveal retina (Figure 5C). Retinal organoids contained 40 cell types (Figure 5D), 32 of which were also present in the adult human retina but eight cell types that were not. We compared these transcriptome-based cell types to known marker-defined cell types or classes (Figures 5E and 5F) using the uncertainty coefficient (*U*). When *U* takes a value of one, the marker-defined cell types explain all of the variability in the transcriptome-based cell types; when *U* takes a value of zero, none of the variability is explained. We assigned significance to an uncertainty coefficient by comparing it to uncertainty coefficients generated by shuffling cluster labels. The highly significant uncertainty coefficients (adult retina: *U* = 0.34, p = 2 × 10^−15^; developed organoid: *U* = 0.25, p = 1 × 10^−12^) suggest correspondence between the transcriptome-based cell types and known marker-defined cell types. We also compared our cell types in more detail to cell-type markers identified in other species (Figure S3). In the case that previously described markers did not strongly and specifically label a cell type in our atlas, we present a new marker for that cell type identified using our data. The topology of transcriptome-based cell types on the UMAP maps was biologically meaningful, with neuronal and non-neuronal cell types arranged on opposite sides, interneurons collected in the bottom left, and bipolar cell types segregating by the polarity of their light response (ON and OFF). Therefore, the distance between two cell clusters on the maps in some cases reflects similarity in function between the cell clusters.

In the adult retina, we detected all known neuronal cell classes as well as many subclasses and distinct cell types, such as rods, L/M (red/green) cones and S (blue) cones, two types of horizontal cells, ten types of bipolar cells in three major groups (rod bipolar, ON cone bipolar, and OFF cone bipolar), 17 types of amacrine cells including glycinergic types (e.g., AII amacrine cells, *GJD2*) and GABAergic types (e.g., starburst amacrine cells, *SLC18A3*), and five types of ganglion cells (Figures 5E, 5H, and S3; Table S2). We detected the following non-neuronal cells: Müller cells, astrocytes, pigment epithelial cells, choroidal melanocytes, microglia, monocytes, natural killer cells, T and B cells, mast cells, pericytes, fibroblasts, and vascular endothelial cells.

In developed organoids, most retinal neuronal cell types and some non-neuronal cell types were present, including rods, S cones and L/M cones, one type of horizontal cells, all ten types of bipolar cells, and 14 of 17 types of amacrine cells found in the adult (Figures 5F, 5H–5J, and S3; Table S2). The only non-neuronal cell types we detected were Müller cells, astrocytes, and pigment epithelial cells. Immune, vascular-associated, and choroidal cell types were not observed in developed organoids. Seven clusters, four of them expressing amacrine cell markers (ACB_02, ACB_05, ACB_06, and ACB_13), containing 1.5% of organoid cells were not present in the adult, indicating some cells had not finished developing.

### Convergence of Cell-Type Transcriptomes of Organoids and Adult Retina

**Figure S4:**
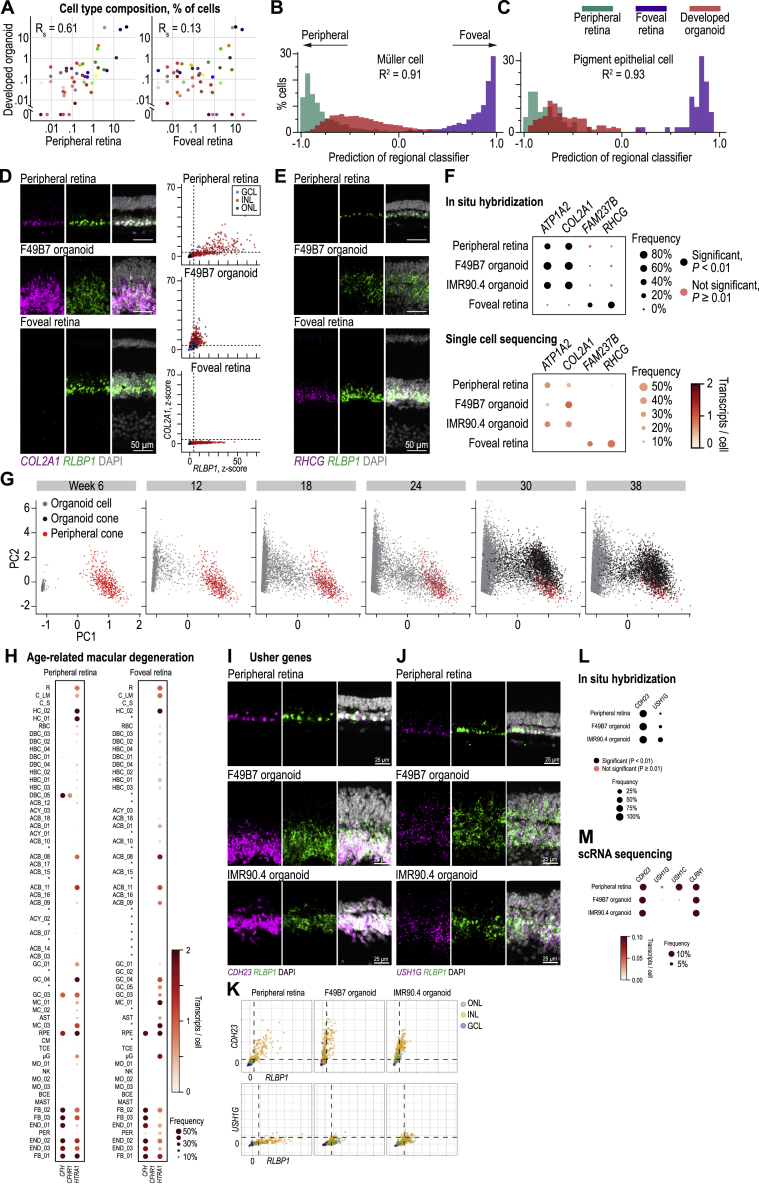
Convergence of Organoid Cell Types to Those of the Adult Peripheral Retina, Cell-Type Expression of Genes Associated with Retinoic-Acid, Age-Related Macular Degeneration, and Usher Syndrome, Related to Figures 5 and 7 and Table S2 (A) Comparison of developed organoid cell type composition to that of peripheral retina (left) and foveal retina (right). Color, cell type as in Figures 5B–5G. RS, Spearman correlation. Cell types shared between organoid and adult retina. (B – C) Predictions from a classifier trained to predict the region of origin (peripheral, −1.0; foveal, +1.0) of (B) Müller cells and (C) pigment epithelial cells based on their transcriptome. Green bars, peripheral cells held out from training; purple bars, foveal cells held out from training; red bars, developed organoid cells; R2, coefficient of determination. (D – E) *In situ* hybridization against regional marker genes. Rows, tissue of origin. (D) Left, confocal images; Magenta, COL2A1 (peripheral Müller cell marker); Green, RLBP1, (Müller cell marker); White, DAPI (nucleus marker). Right, relative marker expression in cells. Points, cells. Color, retinal layer; legend at top right. ONL, outer nuclear layer; INL, inner nuclear layer; GCL, ganglion cell layer. (E) Confocal images. Magenta, RHCG (foveal Müller cell marker); Green, RLBP1; White, DAPI. (F) Comparison of regional marker *in situ* hybridization and transcriptome findings. Rows, tissue / region of origin. Columns, marker genes. Top, *in situ* hybridization. Bottom, single cell RNA sequencing. Dot size, percent of cells expressing. Dot color, mean transcripts per cell. Legends and colormap at right. (G) Comparison of transcriptomes in all organoid cells and adult cones by principal component analysis. Scatterplot axes are the first two principal components (PC1, PC2). Each point is the transcriptome of a cell. From left to right, panels contain cells from organoids of increasing age. Red, peripheral cones; black, developed organoid cones; gray, other organoid cells. (H) Genes from the age-related macular degeneration associated loci 1q31.3 (*CFH* and *CFHR1*, expressed by DBC_05) and 10q26.13 (*HTRA1*, expressed by horizontal cells and types of amacrine and ganglion cells) (Fritsche et al., 2016). Dot size, percent of cells expressing. Dot color, mean transcripts per cell. Right, colormap and legend. Cell type acronyms are from Figures 5B–5G. ^∗^, cell type not present in tissue. (I – M) Genes associated with type I (*USH1G*, *USH1C*, *CDH23*) and type III (*CLRN1*) Usher syndromes which had no consensus for the cell type of origin in the human retina as their expression varies across species (Mathur and Yang, 2015; Sahly et al., 2012; Siegert et al., 2012) are expressed in Müller cells of the adult peripheral and foveal retina and developed organoids. (I – J) Confocal images. *In situ* hybridization. Magenta, *CDH23* or *USH1G* (Usher disease genes); green, *RLBP1* (Müller cell marker); white, DAPI (nuclear marker). (K) Relative marker expression. Points, cells. Color, retinal layer. ONL, outer nuclear layer; INL, inner nuclear layer; GCL, ganglion cell layer. (L – M) Comparison of regional marker (L) *in situ* hybridization and (M) transcriptome findings. Rows, tissue / region of origin. Columns, marker genes. Legends and colormaps at bottom.

Cell-type markers revealed similar cell types in developed organoids and adult retinas but did not provide a quantitative measure of how close the transcriptomes of organoid cell types were to their adult counterparts. To address this, we first asked whether developed organoids resemble more the peripheral or the foveal retina. Using a classifier that we trained on the transcriptomes of cell types in the peripheral and foveal retina, we found that the transcriptomes of developed organoid cells are predominantly peripheral (Figure S4; Table S3). The cell-type composition of developed organoids was also highly and significantly correlated with the peripheral retina but weakly and not significantly correlated with the foveal retina (Figure S4).

**Figure S5:**
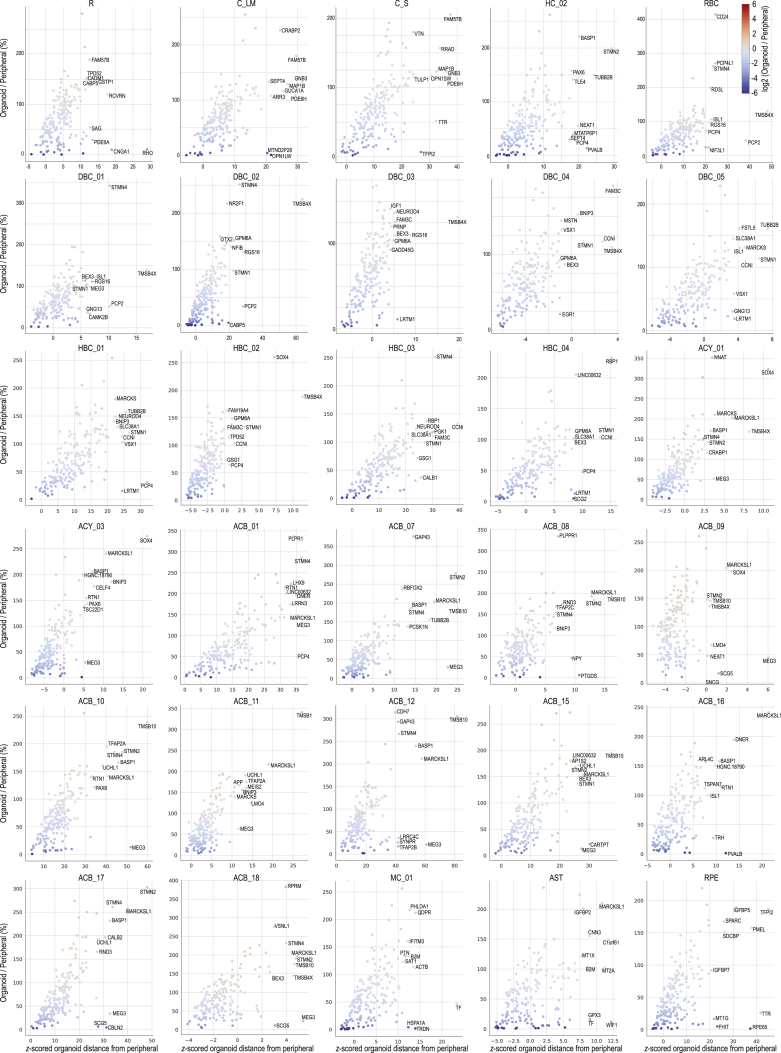
Per Cell-Type Marker Gene Expression Difference between Organoid and Peripheral Retina, Related to Figures 5 and S4 and Table S2 Panels, cell types. Points, top 200 marker genes for each cell type. Labels, top 10 marker genes with the largest expression differences for that cell type. Axes; *x*, distance from organoid gene expression to adult *z*-scored using distribution of differences for the same 200 genes in peripheral retina; *y*, relative expression of gene in organoid compared to peripheral retina. Colormap, log_2_ relative expression of gene in organoid compared to peripheral retina.

**Figure 6:**
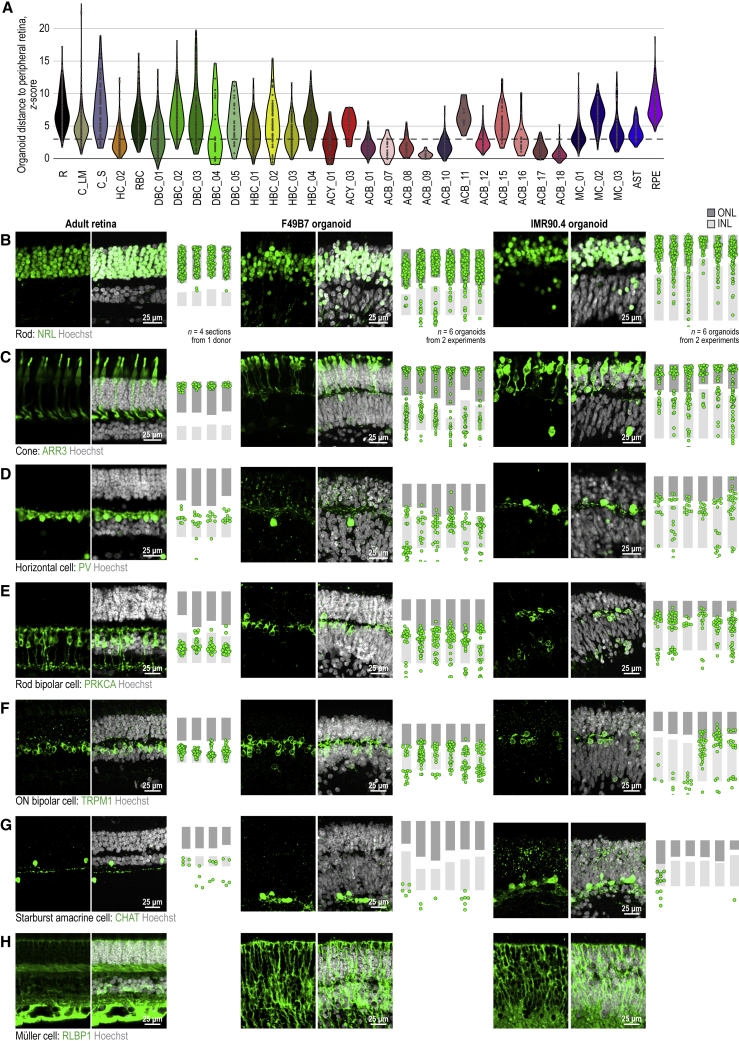
Comparison of Cell Types in Adult Human Retina and Organoids (A) Distance of organoid cell types from the peripheral retina. Distances, *Z* scored Euclidean distance relative to adult cell type’s distribution using 200 cell-type marker genes. Points, single cells. Cell type colors and acronyms according to Figures 5B–5G. (B–H) Cell type markers in adult retinas (left column), F49B7 organoids (middle), and IMR90.4 organoids (right). Left and middle sub-columns, confocal images. White, Hoechst (nucleus marker). Green, antibody against (B) NRL (rod marker); (C) ARR3 (cone marker); (D) PV (horizontal cell marker); (E) PRKCA (rod bipolar cell marker); (F) TRPM1 (ON bipolar cell marker); (G) CHAT (starburst amacrine cell marker); (H) RLBP1 (Müller cell marker). (B–H) Right sub-column, location within retinal layers of cells expressing marker. Points, cells. See also Figures S4, S5, and S6 and Table S2.

We then developed quantitative measures of closeness between groups of developed organoid cells and peripheral retinal cells. For each cell type, we compared developed organoid cells to peripheral retinal cells using the top 200 marker genes for each cell type. On average, some cell type markers had lower expression in organoids than peripheral retina (e.g., organoid rods had 10% of the *RHO* expression observed in the peripheral retina, pigment epithelial cells had 2% *RPE65*) whereas other markers had higher expression (e.g., L/M cones had 226% *CRABP2*, pigment epithelial cells had 158% *PMEL*) (Figure S5). To determine whether individual cells in organoids were within the distribution of marker expression for that cell type in the peripheral retina, we calculated the distance from each cell’s gene expression to the average expression for the cell type in the peripheral retina. We then centered distances and scaled into a *Z* score using the peripheral retina’s distance distribution (Figure 6A). A *Z* score value of zero means the cell’s gene expression has an average distance to the peripheral retina cell type’s average gene expression. Organoid cells with marker expression resembling the peripheral retina cells of the same type should have a *Z* score below three. While the distribution of organoid cell types did not match the adult peripheral retina distribution, all but two cell types (ACB_11 and RPE) had organoid cells overlapping the peripheral retina’s distribution. We also observed overlaps using an alternative comparison based on principal component analysis (Figure S4).

### Post Mortem Changes in Retinal Transcriptome

**Figure S6:**
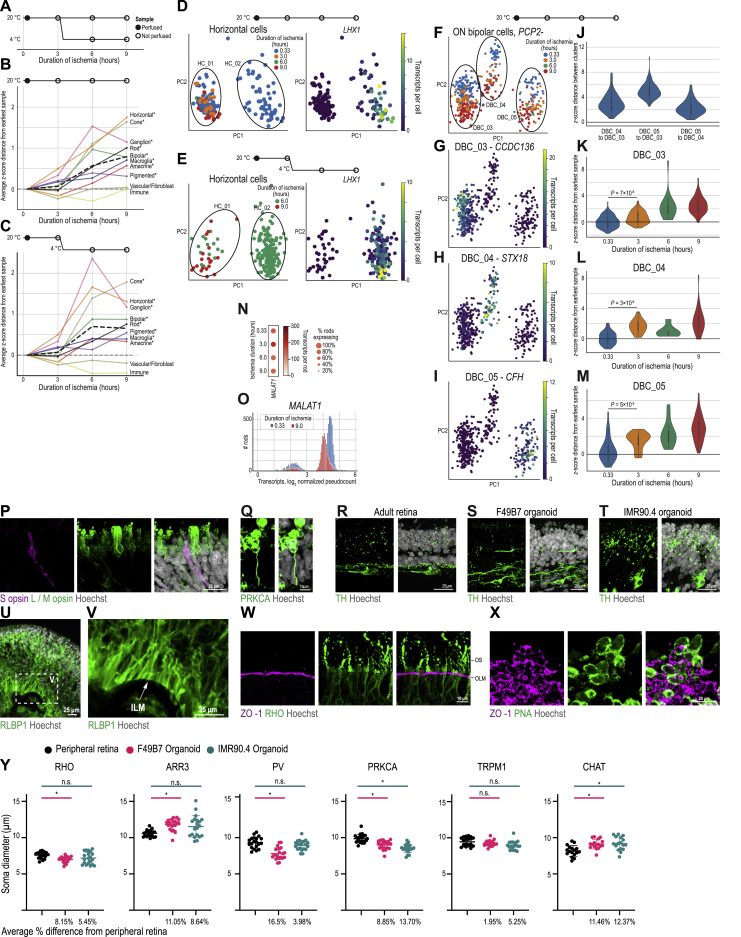
Ischemia Rapidly Alters Gene Expression, Characterization of Cell Types, and Structures in Developed Organoids, Related to Figures 5 and 6 (A) Longitudinal sampling of post mortem human peripheral retina with different durations of ischemia and storage temperatures (n = 2 eyes from 1 donor). Filled circle, sample perfused with oxygenated medium until dissociation. Empty circle, sample not perfused to mimic delayed retrieval. (B – C) Time course of ischemia-induced gene expression changes in cell classes of the (B) retina kept at 20°C, (C) retina transferred to 4°C after three hours. Distance, *z*-scored Euclidean distance relative to the earliest time point (20 minutes) using 200 cell class specific genes. ^∗^, significant change in gene expression (p < 0.01, Mann-Whitney U with Bonferroni correction). (D – E) Principal component analysis of horizontal cells. Color; Left, duration of ischemia; Right, expression of *LHX1* (HC_02 marker). (D) Samples stored at 20°C. (E) Samples moved to 4°C at 3 hours post-mortem. (F – M) ON bipolar cell types not expressing cell type marker *PCP2*. Samples stored at 20°C. (F – I) Principal component analysis of the 3 ON bipolar types not expressing *PCP2*. Color, (F) duration of ischemia; (G) expression of *CCDC136* (DBC_03 marker); (H) expression of *STX18* (DBC_04 marker); (I) expression of *CFH* (DBC_05 marker). (J – M) *z*-scored distances; (J) distance between cell types at 20 minutes. (K – M) gene expression distance versus ischemia duration. Distance, *z*-scored Euclidean distance from the earliest time point. (N) *MALAT1* expression in rods. Rows, duration of ischemia. Right, colormap and legend. (O) Histogram of *MALAT1* expression in rods. Color, duration of hypoxia. (P – X) Confocal images. (P) Magenta, S opsin antibody (blue cone marker); green, L / M opsin antibody (red/green cone marker); white, Hoechst (nucleus marker). (Q) Confocal image. Green, PRKCA antibody; white, Hoechst. (R – T) Green, TH (amacrine cell marker); white, Hoechst. (R) Adult retina, (S) F49B7 Organoid, (T) IMR90.4 Organoid. (U) Green, RLBP1 antibody (Müller cell marker); white, Hoechst. (V) Boxed area from U. ILM, inner limiting membrane. (W) Magenta, ZO-1 antibody (outer limiting membrane marker); green, rhodopsin antibody (RHO, rod outer segment marker). OS, outer segment; OLM, outer limiting membrane. (X) Top view of ZO-1 rings, maximum intensity projection. Magenta, ZO-1 antibody; green, PNA (cone outer segment marker); white, Hoechst. (Y) Quantifications of cell soma diameter of rods (RHO), cones (ARR3), horizontal cells (PV), rod bipolar cells (PRKCA), on bipolar cells (TRPM1) and amacrine cells (CHAT). Dots, cells. Error bars, SD. ^∗^, significant change in soma diameter (p < 0.01, Mann-Whitney U with Bonferroni correction). Data in P – S, U – Y are from F49B7 organoids, data in T and Y from IMR90.4 organoids.

We prioritized a short ischemic window during donor retina collection, assuming that prolonged ischemia could change the retinal transcriptome. To test this hypothesis, we repeatedly single-cell-sequenced pieces of the same human retinas at different time points up to 9 h post-mortem (n = 77,871 cells, n = 2 eyes, from one donor) (Figure S6).

Ischemia induced significant changes in gene expression in rods, cones, horizontal cells, bipolar cells, amacrine cells, ganglion cells, macroglia, and pigmented cells (p < 0.01, Mann-Whitney U test), but we did not detect significant changes in vascular cells/fibroblasts and immune cells (Figure S6). Closely related cell types had different sensitivities to ischemia, for example, type HC_02 horizontal cells could no longer be observed by 3 h at 20°C, whereas type HC_01 horizontal cells were still present at 9 h (Figure S6). Storage conditions made a difference: samples transferred to 4°C retained cell types for longer but only for a period of time (e.g., HC_02 horizontal cells observed at 6 h but not 9 h) (Figure S6). Ischemia-induced transcriptome changes occurred in less than 3 h and were similar in magnitude to the distance between closely related cell types (average *Z* score distance of 9-h sample from 20-min sample; DBC_03, *Z* = 2.3, DBC_04, *Z* = 2.0, DBC_05, *Z* = 2.4; distance from DBC_03 to DBC_04 20-min sample, *Z* = 2.7). *MALAT1* has been proposed as an early marker of rod photoreceptor degeneration (Lukowski et al., 2019), but an increase in the proportion of rods without *MALAT1* expression was not part of the transcriptional changes we observed through 9 h (Figure S6). In summary, we found rapid and strong ischemia-induced changes in gene expression in multiple cell types.

### Morphology of Cell Types in Developed Organoids and Adult Retina

We compared the morphology and localization of cell types in developed organoids with those of the adult retina by immunostaining of markers for different retinal cell types and classes. Many aspects of developed organoid structure were similar to the adult retina. The outer nuclear layer contained cell bodies of rod (Figure 6B) and cone photoreceptors (Figure 6C), with the cell bodies of cones more distal. Both L/M cones and S cones were present (Figure S6). The inner nuclear layer of developed organoids contained horizontal cells (Figure 6D), rod bipolar cells (Figure 6E), ON bipolar cells (Figure 6F), starburst amacrine cells (Figure 6G), dopaminergic amacrine cells (Figure S6), and Müller cells (Figure 6H). Organoids contained some mislocalized cells, compared to the adult peripheral retina. Processes of horizontal cells were elongated horizontally along the outer plexiform layer. PRKCA antibody staining of rod bipolar cells in organoids revealed somatic, dendritic, and axonal signal (Figures 6E and S6). Soma diameters of organoid rods, cones, horizontal cells, rod bipolar cells, ON bipolar cells, and starburst amacrine cells were within 17% of the peripheral retinal soma size, and the average difference was less than 10% (Figure S6). Starburst amacrine cell bodies were arranged at the proximal part of the developed organoid inner nuclear layer or within the ganglion cell layer, matching the adult retina. An inner and outer limiting membrane were present (Figure S6), suggesting that Müller cell processes seal the organoids as they do in the adult retina.

### Disease Map of Cell Types in Developed Organoids and Adult Retina

**Figure 7:**
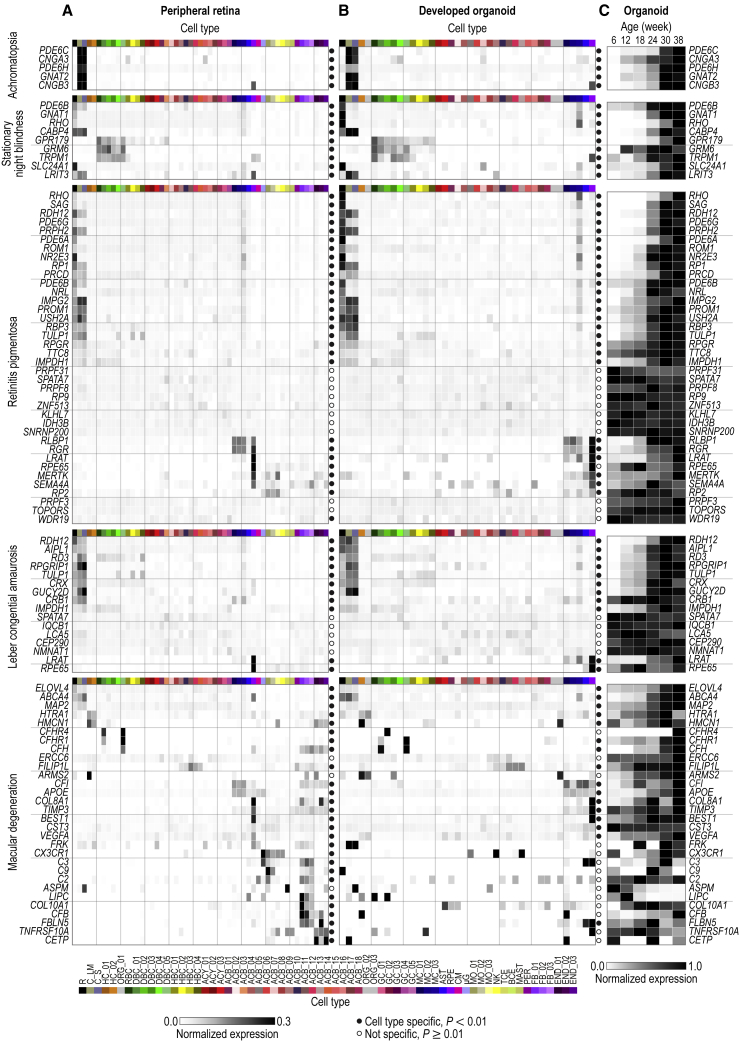
Disease Map for Adult Human Retinas and Developed Organoids (A and B) Normalized expression of disease genes (rows) within cell types (columns) of (A) peripheral retina and (B) developed organoid. Left, names of diseases and associated genes; colormap at bottom left of (A), level of intra-gene normalized expression. Filled circle, gene significantly cell type specific (p < 0.01); empty circle, gene not cell type specific (p ≥ 0.01). Cell type colors and acronyms are according to Figure 5G. (C) Organoid age (columns) dependence of disease gene (rows) expression within organoids. Colormap at bottom, level of min-max normalized expression. Organoid data from F49B7 and IMR90.4 in (B), data from F49B7 organoids in (C). See also Figures S4 and S7 and Tables S2 and S4.

**Figure S7:**
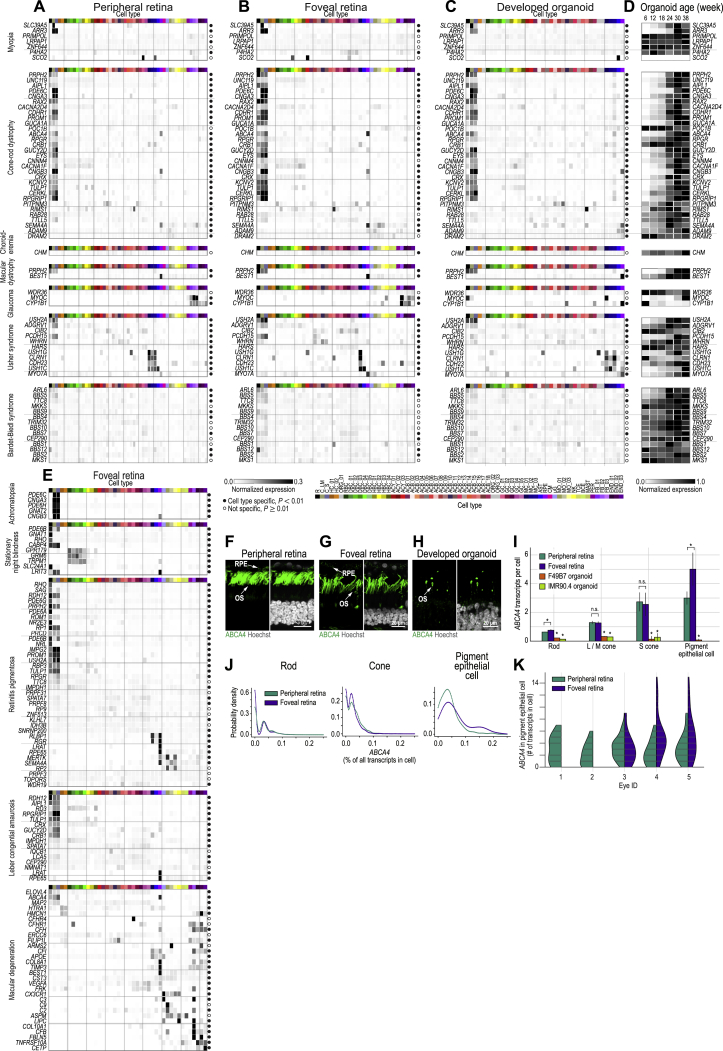
Disease Map for the Peripheral and Foveal Retina and Retinal Organoids; Stargardt Disease Gene ABCA4 Is Overexpressed in Foveal Pigment Epithelial Cells and Rods but Not Cones, Related to Figure 7 and Table S2 (A – E) Disease gene expression in retinal and organoid cell types. (A – C, E) Normalized expression of disease genes (rows) within cell types (columns) in (A) peripheral, (B, E) foveal retina and (C) retinal organoids. Left, names of diseases and associated genes. Colormap at bottom of B, level of intra-gene normalized expression. Filled circle, gene significantly cell type specific (p < 0.01); empty circle, gene not cell type specific (p ≥ 0.01). Cell type colors and acronyms are according to Figures 5B–5G. (D) Age (columns) dependence of disease gene (rows) expression within organoids. Colormap at bottom of D, level of min-max normalized expression. (F – K) Expression of Stargardt disease-associated gene *ABCA4* in photoreceptors and pigment epithelial cells of the peripheral and foveal retina as well as in photoreceptors of developed organoids. (F – H) Confocal images. Green, ABCA4 antibody used in (F) peripheral retina, (G) foveal retina and (H) developed organoid; white, Hoechst (nucleus marker). OS, outer segment; RPE, retinal pigment epithelial cells. F49B7 organoid. (I) Expression level of ABCA4 within cell types and regions in adult retina and developed organoids. Legend, top left. Error bars, ± three SEM. ^∗^p < 1 × 10-2 by Mann-Whitney U with Bonferroni correction. Without a bracket, ^∗^ indicates significant test result versus peripheral and foveal retina. (J) Comparison of peripheral retina (green) and foveal retina (blue) *ABCA4* expression levels in rods, cones, and pigment epithelial cells. Probability densities are generated using a Gaussian kernel density estimate with bandwidth set by Scott’s rule. (K) Violin plots show the *ABCA4* transcript counts in pigment epithelial cells. Expression is subdivided by the eye ID and region of origin. No foveal choroidal / pigment epithelial sample was available for eye IDs one and two. Eye five came from a different donor than eyes three and four.

We mapped genes associated with retinal disease to cell types of the adult retina and developed organoids. We created “disease maps” for 10 non-syndromic retinal diseases for the peripheral (Figures 7A and S7) and foveal retina (Figure S7) as well as for developed organoids (Figures 7B and S7): achromatopsia, congenital stationary night blindness, retinitis pigmentosa, Leber congenital amaurosis, macular degeneration, myopia, cone-rod dystrophy, choroideremia, macular dystrophy, glaucoma, and two syndromic retinal diseases, Usher syndrome and Bardet-Biedl syndrome. We investigated four aspects of these disease maps.

First, we found that most of the diseases considered were cell-type-specific in the peripheral and foveal retina (Figures 7A and S7; Table S4).

Second, while in many cases the retinal cell type expressing the disease-associated gene was the same as that predicted by the clinical phenotype or expression pattern in other species, we found important differences in age-related macular degeneration and in Usher syndrome (Figures S4 and S7).

Third, we compared the disease maps in developed organoids and peripheral retinas (Figures 7A, 7B, and S7; Table S4). The overall pattern of the disease map for most of the considered diseases was similar across developed organoids and the peripheral retina. Because the pathomechanism of the diseases associated with these genes could potentially be studied in patient-derived or gene-edited organoids, we determined the time courses of expression for these genes in organoids (Figures 7C and S7). The timing of expression was variable, with some genes constitutively expressed (e.g., *IQCB1* and *SPATA7*) but others with expression regulated up (e.g., *RHO* and *ABCA4*) or down (e.g., *ASPM* and *CLRN1*) with organoid age.

Fourth, we found differences in the expression of disease genes across peripheral and foveal retinas that might explain the regional specificity of some retinal diseases, such as Stargardt disease (Figure S7).

## Discussion

### Multilayered, Light-Responsive, Synaptically Connected Retinal Organoids

To be useful as a model system for understanding disease mechanisms and for the development of therapies, organoids should contain the relevant features and cell types of their target organ. Organoids with light-responsive photoreceptors have been reported (Zhong et al., 2014), and in developing organoids inner organoid cells were observed to slowly modulate their average activity after light exposure over the course of minutes similar to the developing retina (Hallam et al., 2018). However, we report human organoids capable of transmitting the response of photoreceptors to a flash of light synaptically and on a rapid timescale closer to that observed in the developed eye.

Engineering disease mutations into iPSC lines that form organoids with transcriptomically similar cell types to those of the human retina will allow the study of a wide variety of inherited monogenic diseases as well as the effect of physical or chemical risk factors on specific cell types.

Scaling up the production of organoids with reproducible organ features and transcriptomes will allow screening for the effect of different molecules on organoid cell types. Toward this goal, we described AMASS, a method that allows the generation of 3,700 retinal organoids per single well of iPSCs.

The human retinal organoids we developed reproduced the adult retina’s five-layered structure and aspects of its cell-type diversity and function. However, comparison to the adult retina also revealed morphological, transcriptomic and cell-type compositional differences. In the future, these shortcomings will need to be overcome in order to generate a complete model system of the human retina.

We showed that retinal organoids that we developed are transcriptomically closer to peripheral than to foveal retina. The human foveal cell type transcriptomes we present here can be used to validate studies attempting to induce fovea formation in retinal organoids.

### Functionally Intact Human Retina

A key factor regarding the use of organoids as a model system is the similarity of cell-type transcriptomes in organoids and in the target organ. However, we can only estimate the transcriptomes of cell types in the target organ because we do not have access to them *in vivo*. We found that the transcriptomes of most retinal cell types change rapidly and progressively in ischemic conditions. Therefore, we established a procedure during multi-organ donation to decrease post mortem ischemia to less than 5 min. Our recordings of light responses from 3,550 ganglion cells from these retinas provide evidence of the improved procedure, because post mortem retinas otherwise generally lose this ability. This opens new research avenues; for example, the use of psychophysics to link human visual perception directly to human retinal information processing (Sinha et al., 2017). Light responses have also been recently reported from retinas of eyes removed due to malignancy (Soto et al., 2020).

### Disease Map of Retinal Cell Types

The cell-type transcriptome atlas of developed organoids and adult human retinas allows mapping of disease-associated genes to particular cell types. Single-cell transcriptome atlases for mouse (Macosko et al., 2015; Shekhar et al., 2016) and non-human primate neural retinas (Peng et al., 2019), as well as several hours post mortem human neural retinas, pigment epithelium, and choroid (Kim et al., 2019; Liang et al., 2019; Lukowski et al., 2019; Menon et al., 2019; Peng et al., 2019; Voigt et al., 2019), have been reported. The human atlas from functionally intact, 5-min post mortem neural retinas and from retinal pigment epithelium and choroid described here provides definitive information linking diseases to human cell types. In addition, the reported time-resolved ischemia-induced changes in retinal cell-type transcriptomes can further the understanding of ischemic events in general. Sequencing results, count tables, and atlases are available in the Key Resources Table, Table S2, and at https://data.iob.ch.

Our results indicate that human retinal diseases with genetic associations are cell-type-specific. Therefore, the study of a disease mechanism will be more relevant when performed in the disease-associated cell type. Moreover, the human phenotypes of some genetic diseases do not appear in model organisms such as mice (Veleri et al., 2015). Thus, the species-specific transcriptome in the relevant human cell type and genome likely matters in the pathomechanism. Because cell types of the adult human retina are present in the developed human retinal organoid, it is potentially a model for some retinal diseases. Finally, the mapping of disease genes to cell types has implications for therapy. For example, we found that Stargardt disease (Rivera et al., 2000) may originate in fovea-specific dysfunction of pigment epithelial cells (Lenis et al., 2018). Therapy for Stargardt disease should therefore focus on gene replacement or editing, not only of photoreceptors, but also of pigment epithelial cells. Therapeutic genes could be delivered to relevant cell types using adeno-associated viruses (AAVs) equipped with cell-type-specific promoters (Jüttner et al., 2019).

### Human Retina Atlas as a Reference for Disease States

Post mortem retinas can be obtained not only from individuals without retinal diseases but also from individuals affected by common retinal diseases such as diabetic retinopathy, age-related macular degeneration, and glaucoma. The cell-type transcriptome atlas of adult human retinas described here will serve as ground truth for future description and interpretation of the perturbations in cell-type transcriptomes obtained from patients with retinal diseases. Such comparisons could be the starting point to describe the cell-type-resolved pathomechanism of diabetic retinopathy, age-related macular degeneration, and glaucoma.

## STAR★Methods

### Key Resources Table

**Table:** 

REAGENT or RESOURCE	SOURCE	IDENTIFIER
**Antibodies**		

Mouse monoclonal anti-ABCA4; 1:100	Rockland Immunochemicals	Cat# 200-301-D05; RRID: AB_11181579
Rabbit polyclonal anti-ARL13B; 1:50	Proteintech	Cat# 17711-1-AP; RRID: AB_2060867
Mouse monoclonal anti-Bassoon; 1:800	Enzo	Cat# SAP7F407; RRID: AB_1641480
Goat polyclonal anti-Brachyury; 1:200	R&D Systems	Cat# AF2085; RRID: AB_2200235
Goat polyclonal anti-CHAT; 1:300	Merck Millipore	Cat# AB144P; RRID: AB_2079751
Mouse anti-ARR3 (Cone Arrestin) [7G6]; 1:500	Gift from the Laboratory of Wolfgang Baehr, University of Utah	N/A
Goat anti-ARR3 (Cone Arrestin); 1:200	Novus	Cat# NBP1-37003; RRID: AB_2060085
Mouse monoclonal anti-RLBP1 (CRALBP); 1:400	Abcam	Cat# ab15051; RRID: AB_2269474
Rabbit monoclonal anti-FOXA2; 1:400	Abcam	Cat# ab108422; RRID: AB_11157157
Chicken polyclonal anti-GFP; 1:200	Abcam	Cat# ab13970; RRID: AB_300798
Mouse monoclonal anti-MITF; 1:400	Exalpha Biologicals	Cat# X2398M
Goat polyclonal anti-NANOG; 1:200	R&D Systems	Cat# AF1997; RRID: AB_355097
Mouse monoclonal anti-NCAM (CD56); 1:200	Stem Cell Technologies	Cat# 60021
Rabbit polyclonal anti-NES (Nestin); 1:200	Sigma	Cat# N5413; RRID: AB_1841032
Goat polyclonal anti-NRL; 1:50	Santa Cruz Biotechnology	Cat# sc-10971; RRID: AB_653467
Goat polyclonal anti-NRL; 1:200	R and D Systems	Cat# AF2945; RRID: AB_2155098
Rabbit monoclonal anti-OCT4; 1:100	Abcam	Cat# ab181557; RRID: AB_2687916
Rabbit polyclonal anti-Opsin M / L; 1:400	Merck Millipore	Cat# AB5405; RRID: AB_177456
Goat polyclonal anti-Opsin S; 1:200	Santa Cruz Biotechnology	Cat# sc-14365; RRID: AB_2236550
Mouse monoclonal anti-PVALB (Parvalbumin) clone Parv-19; 1:200	Sigma	Cat# P3088; RRID: AB_477329
Goat polyclonal anti-PRKCA (PKCɑ); 1:500	Santa Cruz Biotechnology	Cat# sc-208-G; RRID: AB_632220
Rabbit monoclonal anti-PRKCA (PKCɑ) [Y123]; 1:200	Abcam	Cat# ab32376; RRID: AB_777294
Mouse monoclonal anti-PRKCA (PKCɑ); 1:100	BD Biosciences	Cat# 610108; RRID: AB_397514
Rabbit polyclonal anti-PSD95	Abcam	Cat# ab18258; RRID: AB_444362
Mouse monoclonal anti-RHO (Rhodopsin) clone 1D4; 1:1000	Sigma	Cat# R5403; RRID: AB_477464
Rabbit polyclonal anti-RIBEYE; 1:800	Synaptic Systems	Cat# 192103
Goat polyclonal anti-SOX17; 1:200	R&D Systems	Cat# AF1924; RRID: AB_355060
Rabbit polyclonal anti-SOX2; 1:200	Merck Millipore	Cat# AB5603; RRID: AB_2286686
Mouse monoclonal anti-SSEA4; 1:200	Invitrogen	Cat# 414000; RRID: AB_1502065
Mouse monoclonal anti-TOMM20; 1:400	Abcam	Cat# ab56783; RRID: AB_945896
Rabbit polyclonal anti-TRPM1; 1:200	Atlas Antibodies	Cat# HPA014779; RRID: AB_2669090
Rabbit polyclonal anti-ZO1; 1:400	Abcam	Cat# ab59720; RRID: AB_946249

**Bacterial and Virus Strains**		

pAAV-EF1a-GCaMP6s-WPRE-pGHpA	Wertz et al., 2015	Addgene Cat# 67526
pUCmini-iCAP-PHP.eB (Rep2-Cap-encoding plasmid)	Chan et al., 2017	Addgene Cat# 103005

**Biological Samples**		

Human retina, male donor	This paper	R-00646_01
Human retina, female donor	This paper	R-00646_03
Human retina, female donor	This paper	R-00646_04
Human retina, male donor	This paper	R-00646_07

**Chemicals, Peptides, and Recombinant Proteins**		

Ames medium	Sigma	Cat# A1420
(±)-CPP (3-[(R)-2-carboxypiperazin-4-yl]-propyl-1-phosphonic acid)	Sigma	Cat# C104
1ml syringe/BD Luer-Lok syringe, with concentric tip	VWR	Cat# 613-2997
3.5 cm cell culture dishes	Milian	Cat# 351008
9-cis-retinal	Sigma	Cat# T0625
96-well Clear Round Bottom Ultra-Low Attachment Microplate	Corning	Cat# 7007
L-AP4 (2-amino-4-phosphonobutyric acid)	Tocris Bioscience	Cat# 0103
Accutase	Thermo Fisher Scientific	Cat# 00-4555-56
Albumin solution human	Sigma	Cat# A9080
B-27 supplement without vitamin A (50 × )	GIBCO	Cat# 12587-010
Benzyl dimethylamine	SERVA Electrophoresis	Cat# 14835.01
Blebbistatin	Sigma	Cat# B0560‒5MG
Bovine serum albumin (BSA)	Sigma	Cat# 05482‒25G
BrainPhys Medium Without Phenol Red	STEMCELL Technologies	Cat# 05791
DMEM Medium	Thermo Fisher Scientific	Cat# 11965-092
DMEM with Glutamax	GIBCO	Cat# #10569-010
DMEM/F-12 Medium	GIBCO	Cat# 31331‒028
DNase	Sigma	Cat# D4263
Dodecenylsuccinic acid anhydride	SERVA Electrophoresis	Cat# 20755.01
DynaBeads MyOne Silane Beads	Life Technologies	Cat# 37002D
EDTA	Invitrogen	Cat# 15575020
F-12 Medium	GIBCO	Cat# 31765‒027
Fetal bovine serum (FBS), Embryonic stem cell qualified	Millipore	Cat# es‒009‒b
Fibroblast Growth Factor-Basic (bFGF)	GIBCO	Cat# PHG0264
Glasgow’s MEM medium	GIBCO	Cat# 21710‒025
Glutaraldehyde	Electron Microscopy Sciences	Cat# 16220
Glycid ether	SERVA Electrophoresis	Cat# 21045.01
Heparin	Sigma	Cat# H3149‒50KU
Hypodermic needles, 27G	VWR	Cat# 613-3834
ImmEdge hydrophobic barrier pen	ACD biotechne	Cat# 310018
Iodixanol density gradient medium OptiPrep	Sigma	Cat# D1556
Lead citrate	Electron Microscopy Sciences	Cat# 17800
Matrigel, growth factor reduced	Corning	Cat# 356230
MEM alpha medium	GIBCO	Cat# 12571
Methylnadic anhydride	SERVA Electrophoresis	Cat# 29452.01
MicroTissues 3D Petri Dish micro-mold spheroids, size L, 9 × 9 array	Sigma	Cat# Z764019-6EA
Millipore Amicon 100K columns	Millipore	Cat# UFC910008
mTesR1 medium	STEMCELL Technologies	Cat# 85850
N-2 supplement (100x)	GIBCO	Cat# 17502‒048
NBQX (2,3-dioxo-6-nitro-7-sulfamoyl-benzo[f]quinoxaline)	Tocris Bioscience	Cat# 1044
Neurobasal-A medium	Thermo Fisher Scientific	Cat# 10888022
Opal 520 dye	Akoya Bio	Cat# FP1487001KT
Opal 570 dye	Akoya Bio	Cat# FP1488001KT
Opal 690 dye	Akoya Bio	Cat# FP1497001KT
Osmium tetroxide	Electron Microscopy Sciences	Cat# 19170
Papain	Worthington Biochemical	Cat# LS003127
Paraformaldehyde (PFA)	Electron Microscopy Sciences	Cat# 15710
Paraformaldehyde (PFA)	Thermo Fisher Scientific	Cat# 28908
PNA-bio, lectin from Arachis hypogaea (peanut)	Sigma	Cat# L6135
Polyethyleneimine	Plysciences	Cat# 24765-1
Primocin	Invivogen	Cat# ant-pm-2
ProLong Gold mounting medium	Thermo Fisher Scientific	Cat# P36934
Propylene oxide	Sigma	Cat# 82320
Retinoic acid	Sigma	Cat# R2625
ROCK inhibitor Y-27632	STEMCELL Technologies	Cat# 72304
Shandon M-1 embedding matrix	Thermo Fisher Scientific	Cat# 1310TS
Sodium cacodylate buffer	Sigma	Cat# 20840
Streptavidin, Alexa Fluor 555 Conjugate	Thermo Fisher Scientific	Cat# S32355
TaqMan Universal PCR Master Mix	Thermo Fisher Scientific	Cat# 4304437
Taurine	Sigma	Cat# T0625‒25G
Thermo Scientific SuperFrost Ultra Plus GOLD Slides	Fisher Scientific	Cat# 11976299
TurboNuclease	Accelagen	Cat# N0103L
UltraPure BSA	Thermo Fisher Scientific	Cat# AM2616
Uranyl acetate	Electron Microscopy Sciences	Cat# 22400
Versene	GIBCO	Cat# 15040066

**Critical Commercial Assays**		

CytoTune-iPS Reprogramming Kit	Invitrogen	Cat# A13780‒01
MycoAlert PLUS Mycoplasma Detection Kit	Lonza	Cat# CABRLT07‒710
DNeasy Blood & Tissue Kit	QIAGEN	Cat# 69504
Vector Blue Alkaline Phosphatase Kit	Vector Laboratories	Cat# SK‒5300
STEMdiff Trilineage Differentiation Kit	STEMCELL Technologies	Cat# 05230
Chromium Single Cell 3′ Library and Gel Bead Kit v2	10x Genomics	Cat# 120237
Chromium Single Cell 3′ Library & Gel Bead Kit v3	10x Genomics	Cat# 1000075
SPRIselect Reagent Kit	Beckman Coulter	Cat# B23317
High Sensitivity DNA Kit for Bioanalyzer	Agilent	Cat# 5067‒4626
Qubit dsDNA high sensitivity (HS) assay kit	Invitrogen	Cat# Q32854
RNAscope Multiplex Fluorescent Reagent Kit v2	ACD biotechne	Cat# 323100
RNAscope 3-plex Negative Control Probe	ACD biotechne	Cat# 320871
RNAscope 3-plex Positive Control Probe	ACD biotechne	Cat# 320861
RNAscope Probe - Hs-RLBP1	ACD biotechne	Cat# 414221
RNAscope Probe - Hs-COL2A1-C3	ACD biotechne	Cat# 427871-C3
RNAscope Probe - Hs-ATP1A2-C3	ACD biotechne	Cat# 497451-C3
ACD New Probe NPR-0004425; FAM237B	ACD biotechne	Cat# 834481-C2
ACD New Probe NPR-0004428; RHCG	ACD biotechne	Cat# 834511-C2
ACD New Probe NPR-0004431; CDH23	ACD biotechne	Cat# 834541-C3
ACD New Probe NPR-0004432; USH1G	ACD biotechne	Cat# 834551-C3

**Deposited Data**		

Bulk transcriptomes of human retina between seven and 20 weeks in developmental age	Hoshino et al., 2017	GEO: GSE104827
Sequencing results related to the cell-type transcriptomes in functional adult human retinas (fovea, periphery and choroid/RPE), ischemic retinas, developing organoids from the iPSC line F49B7 and developed organoids from the iPSC lines F49B7 and IMR90.4	This paper, deposited at European Genome-phenome Archive	EGAS00001004561
Count tables and annotations related to the cell-type transcriptomes in functional adult human retinas (fovea, periphery and choroid/RPE), ischemic retinas, developing organoids from the iPSC line F49B7 and developed organoids from the iPSC lines F49B7 and IMR90.4	This paper, deposited at Mendeley Data	https://doi.org/10.17632/sm67hr5bpm.1

**Experimental Models: Cell Lines**		

iPSC line 01F49i-N-B7 (short name: F49B7)	This paper	01F49i-N-B7
iPSC line iPS(IMR90)-4-DL-01	WiCell	iPS(IMR90)-4-DL-01

**Oligonucleotides**		

Forward primer (PCR for AAV titration): GGAACCCCTAGTG ATGGAGTT	Aurnhammer et al., 2012	N/A
Reverse primer (PCR for AAV titration): CGGCCTCAGTGAGCGA	Aurnhammer et al., 2012	N/A
Probe, 5′–(6FAM) (PCR for AAV titration): CACTCCCTCTCTGC GCGCTCG	Aurnhammer et al., 2012	N/A

**Recombinant DNA**		

pHGT1-Adeno1 helper plasmid	Provided by C. Cepko, Harvard Medical School. Boston, MA, USA	N/A

**Software and Algorithms**		

FIJI	Schindelin et al., 2012	http://www.nature.com/articles/nmeth.2019
Cell Ranger v2.0 / v3.1	10x Genomics	https://support.10xgenomics.com/single-cell-gene-expression/software/downloads/latest
Infomap	Rosvall and Bergstrom, 2008	http://www.pnas.org/cgi/doi/10.1073/pnas.0706851105
LargeVis v0.2.1	Tang et al., 2016	doi:10.1145/2872427.2883041
R: A language and environment for statistical computing.	R Core Team. R Foundation for Statistical Computing. Vienna, Austria.	https://www.R-project.org/
Tidyverse v1.3.0	Wickham et al., 2019	https://joss.theoj.org/papers/10.21105/joss.01686
Points To Curve Distance using Distance Map imageJ Macro	Olivier Burri	https://gist.github.com/lacan/74f550a21ea97f46c74f1a110583586d
GraphPad Prism 8	GraphPad Software	https://www.graphpad.com:443/
CaImAn v1.8.2	Giovannucci et al., 2019	https://elifesciences.org/articles/38173
NumPy v1.14.3	van der Walt et al., 2011	https://numpy.org/
SciPy v0.18.1 / v1.4.1	Virtanen et al., 2020	https://www.scipy.org/
Annoy v1.15.2	Spotify	https://github.com/spotify/annoy
python-igraph v1.1.2	Csardi and Nepusz, 2005	https://igraph.org/python/
scVis	Ding et al., 2018	https://github.com/shahcompbio/scvis
Keras	François Chollet	https://keras.io/
TensorFlow v1.9.0	Google	https://www.tensorflow.org/
scikit-learn v0.19.1	Pedregosa et al., 2012	https://github.com/scikit-learn/scikit-learn
Scrublet	Wolock et al., 2019	https://github.com/AllonKleinLab/scrublet
SCANPY v1.4.6	Wolf et al., 2018	https://github.com/theislab/scanpy
mnnpy	Chris Kang	https://github.com/chriscainx/mnnpy

**Other**		

Website with additional resources associated with the publication	This paper	http://data.iob.ch
Disease maps of cell types in developed organoids and adult retina; fovea, periphery and choroid/RPE (achromatopsia, congenital stationary night blindness, retinitis pigmentosa, Leber congenital amaurosis, macular degeneration, myopia, cone-rod dystrophy, choroideremia, macular dystrophy, glaucoma, Usher syndrome and Bardet–Biedl syndrome)	This paper	Figures 7 and S7
Cell type atlases showing the expression of genes in cell types of the human peripheral retina, human foveal retina, and developed retinal organoid.	This paper	Table S2; https://doi.org/10.17632/sm67hr5bpm.1
NIH Genetics Home Reference	U.S. National Library of Medicine	https://ghr.nlm.nih.gov

### Resource Availability

#### Lead Contact

Further information and requests for resources and reagents should be directed to and will be fulfilled by the Lead Contact, Botond Roska (botond.roska@iob.ch).

#### Materials Availability

All unique/stable reagents generated in this study are available from the Lead Contact with a completed Materials Transfer Agreement.

#### Data and Code Availability

The cell type atlases generated in this study are available as spreadsheets in supplemental data: (i) Developed retinal organoid, library-normalized transcripts per cell; (ii) Adult human peripheral retina, library-normalized transcripts per cell; (iii) Adult human foveal retina, library-normalized transcripts per cell. The count tables generated in this study for the normal and ischemic adult human retina and for F49B7 and IMR90.4 retinal organoids are available on Mendeley Data at https://doi.org/10.17632/sm67hr5bpm.1. Sequencing data has been deposited at the European Genome-phenome Archive (EGA) under accession number EGAS00001004561. The data for bulk RNA sequencing of developing human retina (Hoshino et al., 2017) shown in Figure 3 is available at GEO: GSE104827. The code generated during this study is available upon request to the Lead Contact, Botond Roska (botond.roska@iob.ch). Additional resources are available at https://data.iob.ch.

### Experimental Model and Subject Details

#### Primary human tissue samples

Human retina tissue was obtained from multi-organ donors by sampling non-transplantable eye tissue that was removed in the course of cornea harvesting for transplantation. Donors with a documented history of eye disease were excluded from this study. Personal identifiers were removed and samples were anonymized before processing. All tissue samples were obtained in accordance with the tenets of the Declaration of Helsinki, and experimental protocols were approved by the local ethics committee. The sequencing results include n = 8 eyes from n = 4 donors, donor age was between 50 and 80 years. The donors designated R-00646_01 and R-00646_07 were male and the donors designated R-00646_03 and R-00646_04 were female. Because of sample rarity we could not confidently assess the effects of sex on the results in this study, but we did compare the transcriptomic results between different donors (Figure S2).

#### Human induced pluripotent stem cells

The iPSC line 01F49i-N-B7 (short name: F49B7) is female and was derived from anonymized donor tissue. F49B7 is pluripotent, as evidenced by its expression of pluripotency markers and ability to be directly differentiated into all three germ layers (Figure S1). It also has a normal karyotype, as assessed by G-banding (Cell Guidance Systems, UK) and array comparative genomic hybridization (array CGH; Illumina HumanOmni2.5Exome-8 BeadChip v1.3; LIFE & BRAIN GmbH, Germany).

iPSCs were cultured at 37°C and 5% CO_2_ in a humidified incubator in mTesR1 medium (STEMCELL Technologies, #85850) on 6-well plates (Corning, #3516) coated with Matrigel (Corning, #356230) and passaged in small clumps once per week using Versene (GIBCO, #15040066) or mechanical passaging with a cell-passaging tool (Thermo Fisher Scientific, #23181010) according to WiCell protocols (https://www.wicell.org/stem-cell-protocols.cmsx). Cells tested negative for mycoplasma on a regular basis using the MycoAlert PLUS Mycoplasma Detection Kit (Lonza, #CABRLT07‒710). Cell identity was confirmed by short tandem repeat analysis (STR analysis; Microsynth, Switzerland) on genomic DNA extracted from fibroblasts and iPSCs by the DNeasy Blood & Tissue Kit (QIAGEN, #69504).

### Method Details

#### Cell culture and retinal organoid generation

##### Reprogramming and characterization of induced pluripotent stem cells

Tissue from anonymized donors was received at 4°C in 1 mL ‘fibroblast medium’ containing MEM alpha (GIBCO, #12571), 10% fetal bovine serum (FBS; GIBCO, #26140) and 100 μg / ml Primocin (Invivogen, #ant-pm-2). Biopsy pieces were rinsed with ethanol (70%) and sterile H_2_O and cultured in fibroblast medium, with media changes every other day. After 2 – 3 weeks, fibroblasts were ready to split. At 70% confluency, fibroblasts were cryopreserved in FBS (GIBCO, #26140) supplemented with 20% dimethyl sulfoxide (DMSO; Fisher Scientific, #D128‒500). Fibroblasts were reprogrammed with the CytoTune-iPS Reprogramming Kit (Invitrogen, #A13780‒01) according to the manufacturer’s instructions. In brief, 5 × 10^5^ fibroblasts were plated in one well of a 6-well culture plate with fibroblast medium and infected with Sendai virus at a multiplicity of infection (MOI) of 5:5:3 (KOS, MOI = 5; hc-Myc, MOI = 5; hKlf4, MOI = 3) for 16 h. On day 7, the fibroblasts were plated at 5 × 10^4^ – 1 × 10^5^ onto irradiated mouse embryonic fibroblasts (GIBCO, #S1520‒100). On day 8, the medium was changed to ‘iPSC medium’ containing Glasgow’s MEM (GMEM; GIBCO, #21710‒025), 20% knockout serum replacement (GIBCO, #10828.028), 1% Non-essential Amino Acid Solution (NEAA Solution; Sigma, #M7145), 2 mM GlutaMAX (Thermo Fisher Scientific, #35050038), 55 mM 2-mercaptoethanol (GIBCO, #21985‒023) and 10 μg / mL of fresh Fibroblast Growth Factor-Basic (bFGF; GIBCO, #PHG0264). Once iPSC colonies formed, they were picked, plated on 12-well Corning Synthemax-R plates (Corning, #3979) and transferred during the course of 4 days from iPSC medium to mTesR1 medium (STEMCELL Technologies, #85850) by mixing iPSC medium and mTesR medium (day 1, 25% mTesR; day 2, 50%; day 3, 75%; day 4, 100%).

##### Characterization of human induced pluripotent stem cells

iPSC pluripotency was assessed by staining iPSC colonies cultured in 6-well plates for alkaline phosphatase using the Vector Blue Alkaline Phosphatase Kit (Reactorlab, #SK‒5300), 4 – 6 days after splitting. iPSCs seeded into Matrigel-coated (Corning, #356230) 8-well chamber slides (ibidi, #80826) were further stained for the pluripotency markers SOX2, OCT4, NANOG and SSEA4 (additional antibody information is in the table ‘Key Resources Table’). The potential to differentiate into the three embryonic germ layers was assessed by directed differentiation into ectoderm, mesoderm and endoderm using the STEMdiff Trilineage Differentiation Kit (STEMCELL Technologies, #05230) according to the manufacturer’s instructions on cells maintained in Matrigel-coated (Corning, #356230) 8-well chamber slides (ibidi, #80826). To test for chromosomal aberrations, G-banded karyotyping was performed on 20 cells per line (Cell Guidance Systems, UK). Further, array comparative genomic hybridization (array CGH) was performed using the Illumina HumanOmni2.5Exome-8 BeadChip v1.3 (LIFE & BRAIN GmbH, Germany) on genomic DNA extracted from iPSCs by the DNeasy Blood & Tissue Kit (QIAGEN, #69504).

##### Generation of retinal organoids from human induced pluripotent stem cells

Retinal organoids were generated as described before (Zhong et al., 2014) with some modifications. Of the retinal organoids, 97% contained isolated patches of pigment epithelium at week 38 (n = 135 organoids, n = 3 experiments). The photoreceptors in 97% of organoids grew processes resembling outer segments (Figure 1L), which at week 32 had a length of 47 ± 7.5 μm (mean ± SD, n = 31 organoids, n = 4 experiments).

##### Generation of embryoid bodies without size regulation

On day zero of differentiation, floating embryoid bodies (EBs) were generated by dissociating iPSC colonies from one well of a 6-well plate (Corning, #3516) into small colony pieces with a cell-passaging tool (Thermo Fisher Scientific, #23181010). The EBs were cultured in suspension in mTesR1 medium supplemented with 10 μM blebbistatin (Sigma, #B0560‒5MG) on 3.5-cm untreated Petri dishes (Corning, #351008). On days 1 and 2, one third of the medium was exchanged with ‘neural induction medium’ (NIM) containing DMEM / F12 (GIBCO, #31331‒028), 1 × N2 Supplement (GIBCO, #17502‒048), 1% NEAA Solution (Sigma, #M7145) and 2 μg / ml heparin (Sigma, #H3149‒50KU). On day 3, to remove dead cells and debris, EBs were sedimented by gravity in a 15 mL tube, washed with NIM, and cultured in a 3.5-cm untreated Petri dish (Corning, #351008) in NIM. Half of the NIM was exchanged daily. On day 7, EBs from one 3.5-cm dish were plated onto a 6-cm dish (Corning, #430166) coated with Growth Factor-Reduced Matrigel (Corning, #356230) and then maintained with daily NIM changes.

##### Generation of embryoid bodies in agarose microwell arrays to regulate size

Agarose microwell arrays were prepared in ‘MicroTissues 3D Petri Dish micro-mold spheroids’ molds (size L, 9 × 9 array; Sigma, #Z764019-6EA) with 500 μl of 2% agarose (Thermo Fisher Scientific, #R0491) in DMEM with GlutaMax (GIBCO, #10569-010). The solidified molds were transferred to 12-well plates (Corning, #3513) and equilibrated with 1.5 mL mTeSR medium. The molds were stable for several weeks at 4°C and were warmed in the incubator at 37°C before use. To seed iPSCs into the agarose microwell arrays, a well of iPSCs was washed twice with 1 mL PBS without CaCl_2_ and MgCl_2_ (GIBCO, #14190094), incubated for 5 minutes with 600 μl 0.5 mM EDTA working solution (0.5M EDTA, Invitrogen, #15575020 in PBS without CaCl_2_ and MgCl_2_) at 37°C after which EDTA was aspirated. 500 μl of Accutase (Thermo Fisher Scientific, #00-4555-56) were added, the plate incubated for 3 minutes at 37°C, 1 mL mTeSR medium was added and the cells were gently pipetted up and down to generate a single cell suspension for cell counting. 5 mL of mTeSR medium was added, the cells were pelleted for 5 minutes at 200 g, resuspended in the appropriate working volume and cells were seeded at 150 to 3,000 cells per microwell (each microwell array mold contains 81 microwells) in 150 μl mTeSR containing 10 μM ROCK inhibitor Y-27632 (STEMCELL Technologies, #72304) into the top of the agarose mold. The plates were placed in the incubator for 30 minutes to allow the cells to settle into the microwells before gently filling up the well with 1.5 mL mTeSR medium containing 10 μM ROCK inhibitor to completely cover the agarose mold and incubating the forming embryoid bodies at 37°C, 5% CO_2_. The medium was replaced with NIM on the following schedule: on day one, one third was replaced; on day two, one half was replaced; on day three, all of the medium was replaced. From day four to six embryoid bodies were fed daily with 1.5 mL of NIM and on day 7 embryoid bodies from one well were transferred to a 6-well plate coated with Matrigel (Corning, #356230).

Traditionally, the control of embryoid body size has been achieved by seeding many thousand dissociated iPSCs per well in low attachment 96-well plates (Kuwahara et al., 2015; Nakano et al., 2012). However, we achieved optimal embryoid body size by seeding less than 1,000 iPSCs per microwell. Seeding this low number of iPSCs into 96-well plates does not efficiently generate embryoid bodies. Interestingly, we found that the efficacy of organoid production was more sensitive to changes in embryoid body size using IMR90.4 iPSCs than F49B7 iPSCs, suggesting that embryoid body size should be optimized for each line.

For embryoid bodies from both generation methods, on day 16, NIM was exchanged for ‘3:1 medium’ containing 3 parts DMEM (GIBCO, #10569‒010) per 1 part F12 medium (GIBCO, #31765‒027), supplemented with 2% B27 without vitamin A (GIBCO, #12587-010), 1% NEAA Solution, 1% penicillin / streptomycin (GIBCO, #15140‒122). On day 28 – 32 retinal structures were detached from the Matrigel plate by either microdissection (Zhong et al., 2014) or by checkerboard scraping. Microdissection of neuroretinal structures was performed with needles (VWR, #613-3834) held by a syringe (VWR, #613-2997) under an inverted microscope (EVOS XL Core, Thermo Fisher Scientific, #AMEX1000) in a cell culture hood. Retinal structures were cultured in low attachment 3.5-cm dishes (Milian, #351008) in 3 mL medium.

For checkerboard scraping, first, to break the tissue sheets into smaller pieces, a 1 to 2 mm^2^ grid was scratched through the cells on the culture plate with a 10-μl or 200-μl pipette tip. Second, the entire contents of the culture plate were washed off the plate with a 1,000-μl pipette tip to generate numerous retinal aggregates (Video S1) and small uncharacterized debris. During the manual microdissection step in traditional organoid production, many retinal structures could not be identified by the observer and were consequently left behind, hence checkerboard scraping increased the yield of retinal structures that could be harvested. Retinal structures were typically not broken by the pipette passing through the dish, but rather came off the plate as large tissue pieces. The aggregates contained both regions of neural retina and retinal pigment epithelium. To remove debris and single cells, the aggregates were washed 3 × in a 15 mL tube by sedimentation in 3:1 medium and then maintained in suspension on sterile Petri dishes (VWR, #391‒2016) in 10 – 15 mL 3:1 medium with media changes every other day. Aggregates without phase-bright, stratified neuroepithelium indicative of retina formation (Figure S1) were sorted out one week after checkerboard scraping to leave behind only high-quality retinal organoids. Even before sorting, up to 80% of aggregates within the plate contained phase-bright, stratified neuroepithelium. Organoids were not further trimmed to remove non-retinal structures, because the presence of non-retinal tissue did not prevent growth and maturation of retinal tissue within the organoid.

From day 42, aggregates were cultured in 3:1 medium supplemented with an additional 10% heat-inactivated FBS (Millipore, #es‒009‒b) and 100 μM taurine (Sigma, #T0625‒25G) with media changes every other day. At week 10, the culture medium was supplemented with 1 μM retinoic acid (Sigma, #R2625). From week 14, the B27 supplement in 3:1 media was replaced by N2 supplement (GIBCO, #17502‒048) and retinoic acid was reduced to 0.5 μM.

#### Histology

##### Fixation of human retina and retinal organoids

Human retina tissue was fixed overnight at 4°C in 4% weight / volume (wt / vol) paraformaldehyde (PFA) in phosphate buffered saline (PBS). Organoids were fixed for 4 hours at 4°C in 4% PFA in PBS. After fixation, samples were washed 3 × 30 minutes with PBS and cryopreserved in 30% sucrose in PBS overnight at 4°C. Samples were stored at −80°C until use.

##### Preparation and staining of cryosections

Cryosections (20 – 40 μm) were generated using a cryostat (MICROM International, #HM560) on organoids and human retina embedded in O.C.T compound (VWR, #25608‒930). Sections were mounted onto Superfrost Plus slides (Thermo Fisher Scientific, #10149870), dried for 4 to 16 hours at room temperature and stored at −80°C until use. Photoreceptor outer segments in retinal organoids were not preserved upon OCT embedding. Therefore, for cryosectioning of organoids with preserved photoreceptor outer segments, organoids were embedded in 7.5% gelatin and 10% sucrose in PBS (Lancaster and Knoblich, 2014b).

For immunostainings of cryosections, slides were first dried for 30 minutes at room temperature and then rehydrated for 5 – 10 minutes in PBS. Second, slides were blocked with ‘blocking buffer A’ which was PBS supplemented with 10% normal donkey serum (Sigma, #S30‒100ML), 1% (wt / vol) bovine serum albumin (BSA; Sigma, #05482‒25G), 0.5% Triton X-100 in PBS (Sigma, #T9284‒500ML) and 0.02% sodium azide (Sigma, #S2002‒25G) at room temperature for 1 h. Sections were then incubated in a humidified chamber with primary antibodies (see table, ‘Key Resources Table’). For each slide, primary antibodies were diluted in 100 μl of ‘blocking buffer B’ which was PBS supplemented with 3% normal donkey serum, 1% BSA, 0.5% Triton X-100 in PBS and 0.02% sodium azide overnight at room temperature. After washing 3 × 15 minutes in PBS with 0.1% TWEEN 20 (Sigma, #P9416‒100ML), slides were incubated with secondary antibodies (Thermo Fisher Scientific, donkey secondary antibodies conjugated to Alexa Fluor 488, 568 or 647) diluted 1:500 in 100 μl blocking buffer B for two hours at room temperature in the dark. The sections underwent washes of 2 × 15 minutes in PBS with 0.1% Tween, one wash of 15 minutes in PBS, and were coverslipped with ProLong Gold (Thermo Fisher Scientific, #P36934). Images were acquired with an LSM 700 confocal microscope (Zeiss) or a spinning disc microscope (Axio Imager M2 upright microscope, Yokogawa CSU W1 dual camera T2 spinning disk confocal scanning unit, Visitron VS-Homogenizer or an Olympus IXplore Spin confocal spinning disc microscope system).

Image analysis and quantifications were performed in Fiji (Schindelin et al., 2012).

##### Preparation and staining of vibratome sections

For Vibratome sectioning (VT1000S vibratome, Leica), organoids were embedded in 3% agarose (Promega, #V3125), sectioned at 120 μm thickness and collected in 30% sucrose in 1 × PBS for cryopreservation. Sections were stored at −80°C until use.

Vibratome sections were stained floating in 24-well plates by incubating for 5 hours in blocking buffer A which was PBS supplemented with 10% normal donkey serum (Sigma, #S30‒100ML), 1% (wt / vol) bovine serum albumin (BSA; Sigma, #05482‒25G), 0.5% Triton X-100 in PBS (Sigma, #T9284‒500ML) and 0.02% sodium azide (Sigma, #S2002‒25G) at room temperature followed by incubation in 120 μl primary antibody solution diluted in blocking buffer B which was PBS supplemented with 3% normal donkey serum, 1% BSA, 0.5% Triton X-100 in PBS and 0.02% sodium azide for 3 to 6 days at 4°C. For vibratome sections, the antibody concentrations were doubled compared to those described for cryosections. Sections were washed 3 × in PBS supplemented with 0.05% Triton X-100 (PBS-T) for a total of 24 h, incubated with secondary antibodies (Thermo Fisher Scientific, donkey secondary antibodies conjugated to Alexa Fluor 488, 568 or 647), diluted 1:250 in blocking buffer B for 4 hours at room temperature and washed 3 × in PBS-T for a total of 24 h. Sections were transferred to glass slides using a paint brush and mounted using ProLong Gold (Thermo Fisher Scientific, #P36934). Images were acquired using a spinning disc microscope (Nikon Ti2-E Eclipse inverted, Yokogawa CSU W1 dual camera T2 spinning disk confocal scanning unit, Visitron VS-Homogenizer).

##### Staining of unsectioned organoids

Wholemount staining of organoids was performed by incubating fixed organoids for 3 days in blocking buffer A which was PBS supplemented with 10% normal donkey serum (Sigma, #S30‒100ML), 1% (wt / vol) bovine serum albumin (BSA; Sigma, #05482‒25G), 0.5% Triton X-100 in PBS (Sigma, #T9284‒500ML) and 0.02% sodium azide (Sigma, #S2002‒25G) at room temperature, followed by incubation in 100 μl primary antibody solution diluted in blocking buffer B which was PBS supplemented with 3% normal donkey serum, 1% BSA, 0.5% Triton X-100 in PBS and 0.02% sodium azide for 5 days at 4°C. For wholemount staining, the antibody concentrations were double those described for cryosections. Organoids were washed 3 × in PBS supplemented with 0.05% Triton X-100 for a total of 3 days at room temperature, incubated with secondary antibodies (Thermo Fisher Scientific, donkey secondary antibodies conjugated to Alexa Fluor 488, 568 or 647), diluted 1:250 in blocking buffer B for 4 days at 4°C and washed 3 × in PBS-T for a total of 4 days. Organoids were imaged on an inverted spinning disk confocal microscope (Nikon Ti2-E Eclipse Inverted motorized stand + Yokogawa CSU W1 Dual camera T2 spinning disk confocal scanning unit. Visitron VS-Homogenizer) in 8-well chamber slides (ibidi, #80826).

##### Electron microscopy

Organoids were fixed in 2.5% glutaraldehyde (Electron Microscopy Sciences, #16220) and 2% PFA (Electron Microscopy Sciences, #15710) in 0.1 M sodium cacodylate buffer (Sigma, #20840) overnight at 4°C, washed with 0.1 M cacodylate buffer, and post-fixed in 1% osmium tetroxide (Electron Microscopy Sciences, #19170) for 2 hours at room temperature. Organoids were then dehydrated in a graded ethanol (Sigma, #51976) series (25%, 50%, 75%, and 100%), further dehydrated in propylene oxide (Sigma, #82320) and embedded in Epon resin (SERVA Electrophoresis; glycid ether #21045.01, dodecenylsuccinic acid anhydride #20755.01, methylnadic anhydride #29452.01 and benzyl dimethylamine #14835.01) for 12 h. Semi-thin (0.4 μm) and ultra-thin sections (50 nm) were cut with a Leica EM UC7 ultramicrotome and the latter were collected on formvar-coated single-slot copper grids (Electron Microscopy Sciences, #FF2010-Cu) for imaging. Sections were contrasted with 1% uranyl acetate (Electron Microscopy Sciences, #22400) and lead citrate (Electron Microscopy Sciences, #17800) (8 minutes each) and imaged on a FEI Tecnai Spirit electron microscope (FEI Company) operated at 120 kV using a side-mounted 2K × 2K CCD camera (Veleta, Olympus).

##### In situ hybridization

Human retinal tissue was dissected in oxygenated Ames medium using fine scissors, fixed overnight in 4% (wt / vol) paraformaldehyde (Thermo Fisher Scientific, #28908, in PBS) and washed with PBS for 48 hours at 4°C. Subsequently, 5 × 5 mm retinal pieces were isolated and cryoprotected with 30% (wt / vol) sucrose, before being embedded in Shandon M-1 embedding matrix (Thermo Fisher Scientific, #1310TS). Each retinal piece was cryosectioned into 15 μm thick sections (CryoStar NX70 Cryostat, Thermo Fisher Scientific, #957000), dried at 60°C for 2 hours and immediately stored at −80°C until use.

Organoids were fixed in freshly prepared 4% (wt / vol) paraformaldehyde (Thermo Fisher Scientific, #28908) in PBS overnight at 4°C. Organoids were washed in PBS and cryopreserved by sequentially incubating in 10%, 20% and 30% (wt / vol) sucrose at 4°C until the organoid sunk to the bottom of the tube. Organoids were then immediately embedded in O.C.T Compound (VWR, #25608‒930), frozen on dry ice and stored wrapped in tin foil inside of a zip lock bag at −80°C. 18 μm serial cryosections were prepared on a Cryostat (MICROM International, #HM560), collected onto slides (SuperFrost Ultra Plus GOLD slides, Fisher Scientific, #11976299) and immediately placed on a 60°C hot plate for 2 hours to enhance tissue attachment. Slides were stored at −80°C until use.

RNA was detected in sections of human fovea, periphery and retinal organoids by using the RNAscope Multiplex Fluorescent Reagent Kit v2 Assay (ACD biotechne, #323100) according to the user Manual (Section, ‘Fixed-frozen tissue samples’) with small modifications.

In brief, the slides were first incubated for 30 minutes at 60°C in the ACD HybEZ II Hybridization oven to dry the sections. The sections were then rehydrated for five minutes in PBS, post fixed for 15 minutes in fresh 4% Paraformaldehyde (Thermo Fisher Scientific, #28908) in PBS at 4°C, washed three times two minutes in PBS and finally dehydrated by immersing in 50%, 75% and twice in 100% Ethanol (Sigma, #1009831000) for five minutes each. After drying the sections for five minutes, they were incubated with five to eight drops of RNAscope Hydrogen Peroxide for 10 minutes at room temperature and washed with distilled water. Antigen retrieval was performed in a steamer by first incubating the slides for 10 s in distilled water and then for five minutes in RNAscope 1 × Target Retrieval Reagent, both at 99°C. The slides were then rinsed in distilled water, incubated for three minutes in 100% ethanol and dried at room temperature for five minutes. Afterward a hydrophobic barrier was drawn around the sections using the ImmEdge hydrophobic barrier pen (ACD biotechne, #310018). The slides were then incubated with five drops of Protease III for 10 minutes at 40°C. Longer incubation times led to poor tissue integrity. After washing twice with distilled water the sections were incubated with probes (C1, C2 and C3 probes on the same slide as well as positive and negative control probes on additional two slides, refer to Key Resources Table for information on the probes) for two hours at 40°C. Slides were washed with RNAscope wash buffer twice for two minutes and stored in 5 × Saline Sodium Citrate (SSC; 20 × stock solution containing 3 M sodium chloride and 300 mM trisodium citrate, pH 7.0) at room temperature overnight. On the next day, slides were washed twice with wash buffer and for signal amplification incubated with RNAscope FL v2 Amp1 for 30 minutes at 40°C, Multiplex FL v2 Amp 2 for 30 minutes at 40°C and Multiplex FL v2 Amp 3 for 15 minutes at 40°C with two two-minute washes in wash buffer in between each step. To develop the fluorescent signal, slides were incubated with Multiplex FL v2 HRP-C1 for 15 minutes at 40°C, with Opal 520 dye (Akoya Bio, #FP1487001KT) diluted 1:1500 in TSA buffer (provided with the Multiplex FL v2 kit) for 30 minutes at 40°C and with Multiplex FL v2 HRP blocker for 15 minutes at 40°C with two two-minute washes in wash buffer after each step. The other two channels were developed in the same way, using Multiplex FL v2 HRP-C2 and Multiplex FL v2 HRP-C3 for the respective channels and Opal 570 dye (Akoya Bio, #FP1488001KT) or Opal 690 dye (Akoya Bio, #FP1497001KT) as a fluorescent label. Finally, DAPI solution provided by the kit was added to the slides for 30 s and the slides were coverslipped using ProLong Gold Antifade Mountant (Thermo Fisher Scientific, #P36934). Images were acquired using an Olympus FV3000-BX63L upright cLSM confocal microscope using a 40 × oil objective (Olympus UPlanFL N 40 × , numerical aperture 1.30), the image acquisition settings were adjusted using the positive and negative control slides and subsequently used for all imaging of test probes.

#### Measuring synaptic transmission in retinal organoids

##### AAV production and titration

The design and production of AAVs (adeno-associated virus) has been described previously (Jüttner et al., 2019). Briefly, HEK293T cells were incubated in 10 15-cm culture dishes, in 25 mL of DMEM (Thermo Fisher Scientific, #11965-092) containing 7 μg AAV transgene plasmid (pAAV-EF1a-GCaMP6s-WPRE-pGHpA; Addgene, #67526) (Wertz et al., 2015), 7 μg Rep2-Cap-encoding plasmid (PhP.eB; Addgene, #103005) (Chan et al., 2017), 20 μg pHGT1-Adeno1 helper plasmid (provided by C. Cepko, Harvard Medical School. Boston, MA, USA) and 6.8 μM polyethyleneimine (Polysciences, #24765-1). Cells were collected after 60 hours and resuspended in a solution containing 150 mM NaCl and 20 mM Tris-HCl, pH 8.0. Cell-lysis was obtained by repeated freezing-thawing cycles. Finally, 1 mM of MgCl_2_ was added to the solution. Subsequently, DNA (both plasmid and genomic) was removed by incubation with 250 U / ml of TurboNuclease (Accelagen, #N0103L) at 37°C for 10 minutes. The solution was centrifuged at 4,000 × g. for 30 minutes and the AAV particles in the supernatant were isolated by applying a discontinuous iodixanol density gradient medium (OptiPrep; Sigma, #D1556). The solution was then ultra-centrifuged at 242,000 × *g* for 90 minutes. The concentrated AAV particles were then purified in Millipore Amicon 100K columns (Millipore, #UFC910008). For titration, AAV particles were denatured by treatment with protease K (Fisher Scientific, #10103533) and the encapsidated viral DNA quantification was performed with TaqMan (TaqMan Universal PCR Master Mix; Thermo Fisher Scientific, #4304437) reverse transcription PCR (forward primer: 5′–GGAACCCCTAGTGATGGAGTT; reverse primer: 5′–CGGCCTCAGTGAGCGA; probe: 5′–(6FAM) CACTCCCTCTCTGCGCGCTCG) (Aurnhammer et al., 2012). The final titer measured 4.37 × 10^13^ genomic copies / ml.

##### AAV infection of human retinal organoids

F49B7 retinal organoids were infected at week 35 with AAV (serotype, PhP.eB; Addgene, #67526) inducing the expression of the calcium sensor GCaMP6s under the control of the generic promoter EF1α.

Individual organoids were placed in a single well of an ultra-low attachment U-bottom 96-well plate (Corning, #7007) and maintained at 37°C in 5% CO_2_ in 27 μl culturing media and 3 μl AAV. After 5 hours, 70 μl of fresh media was added to each well. One day later, 100 μl of fresh media was added to each well. After 24 hours, and every 48 hours thereafter the solution was completely exchanged with fresh media.

##### Culture of AAV-infected retinal organoids

Infected organoids were cultured for 3 to 4 weeks in either DMEM (GIBCO, #10569-010) supplemented with 20% Ham’s F12 Nutrient Mix (GIBCO, #31765-027), 10% heat-inactivated fetal bovine serum (Millipore, #es‒009‒b), 1% N2 Supplement (GIBCO, #17502-048), 1% NEAA Solution (Sigma, #M7145), 100 μM taurine (Sigma, #T0625), and 1 μM retinoic acid (Sigma, #R2625) or in BrainPhys media (STEMCELL Technologies, #05791) supplemented with 1% N2 Supplement (GIBCO, #17502-048), 100 μM taurine (Sigma, #T0625), 1 μM retinoic acid (Sigma, #R2625). Two days before calcium imaging recordings, organoids were gradually transferred to BrainPhys media (StemCell Technologies, #05791) supplemented with 1% N2 Supplement (GIBCO, #17502-048), 100 μM taurine (Sigma, #T0625), 1 μM retinoic acid (Sigma, #R2625), 10 μM 9-cis-retinal (Sigma, #R5754), and 2 μM Albumin solution human (Sigma, #A9080).

##### Calcium imaging of organoid light responses

Imaging was carried out using a two-photon microscope (Femtonics, FEMTOSmart Resonance Scanning 2P, SN20170097006). Shortly before recording, the retinal organoids were embedded in 1% low-melting agarose (Thermo Fisher Scientific, #16520050) and placed in a bath imaging chamber (Warner Instruments, #RC-26G). Throughout the recording, the organoid being studied was perfused with BrainPhys media (STEMCELL Technologies, #05791) supplemented with 1% N2 Supplement (GIBCO, #17502-048), 100 μM taurine (Sigma, #T0625), 1 μM retinoic acid (Sigma, #R2625), 10 μM 9-cis-retinal (Sigma, #R5754), 2 μM Albumin solution human (Sigma, #A9080). The perfusion solution was flowing at a rate of 1 – 2 mL / minute, maintained at a temperature of 37°C and bubbled with 95% O_2_ / 5% CO_2_. GCaMP6s expressing cells were imaged at 980 nm (Spectra-Physics, inSight X3) and fluorescence emission was viewed through an immersion objective lens (16 × , NA 0.8, Nikon). The imaging window was set to 200 μm × 200 μm and the *z*-plane was adjusted to show a cross section of the organoid, including all layers (outer nuclear, inner nuclear and ganglion cell) of the retinal tissue including photoreceptor outer segments.

For pharmacological experiments, glutamatergic synaptic transmission was blocked with a mixture of 10 μM NBQX (2,3-dioxo-6-nitro-7-sulfamoyl-benzo[f]quinoxaline; Tocris Bioscience, #1044), 10 μM ABP (2-amino-4-phosphonobutyric acid; Tocris Bioscience, #0103), 10 μM (±)-CPP (3-[(R)-2-carboxypiperazin-4-yl]-propyl-1-phosphonic acid; Sigma, #C104) applied through the perfusion system. Recording was performed 30 minutes after pharmacology administration.

##### Two photon stimulation

To stimulate photosensitive cells, the same laser used for two-photon imaging was repeatedly shuttered for 20 s and un-shuttered for 4 s during imaging (n = 12 trials). This is equivalent to recording during the ‘light onset’ window of a step stimulus. As a control, cells were imaged continuously for 5 minutes, providing a constant background level of light stimulation.

#### Human retinal tissue handling

Eye enucleation was performed while circulation and ventilation were still in place. The enucleated eye was opened and the eye cup flattened with butterfly cuts. The vitreous was then removed. The tissue was submerged in flowing Ames medium (Sigma, #A1420), saturated with carbogen gas (95% O_2_, 5% CO_2_). The time elapsed from central retinal artery clamp to artificial *ex vivo* perfusion was less than 5 minutes for all eyes except the eye used to study the effects of ischemia on the retinal transcriptome. Samples intended for electrophysiology were subsequently handled in a dark room using dim red illumination for general dissection and night vision (ATN Corp, USA) with infrared illumination for dissection under a microscope. The regions of the retina sampled for sequencing were: (i) peripheral retina; squares 2 – 3 mm on a side; ventral midline at 50% eccentricity (midway between the fovea and ventral retina border with the ora serrata) (ii) foveal retina; disc 1.5 mm in diameter; centered on the fovea centralis. Landmarks used to locate the fovea included (i) adjacency to the optic disc, (ii) the avascular zone, (iii) pigmentation of the macula lutea, and (iv) retinal transparency at the fovea centralis due to retinal thinning. In both regions, the neural retina tissue was separated from the retinal pigment epithelium / choroid and the two tissues were dissociated into single cells for analysis separately. To study ischemia, peripheral retina was immediately removed from the sclera and sectioned into pieces. One piece from each retina was transferred to perfusion until dissociation and, to mimic delayed retrieval, the other pieces were stored with vitreous media at room temperature (20°C). After three hours, half of the non-perfused samples were transferred to 4°C to study whether storing samples cooled would influence the transcriptome.

#### Single-cell RNA sequencing of retinal organoids and human retina

##### Dissociation of organoids and human retina into single cells

The retinal region of organoids, which could be clearly distinguished in light microscopy, was dissected from the main body of the organoid using needles. The single-cell dissociation protocol was adapted from an existing protocol (Balse et al., 2005). The retinal piece (derived from human retina or retinal organoid) was washed 2 × at 37°C with 1 mL ‘ringer solution’ without calcium containing 125.6 mM NaCl, 3.6 mM KCl, 1.2 mM MgCl_2_·H_2_O, 22.6 mM NaHCO_3_, 21.7 μM NaH_2_PO_4_·H_2_O, 70.2 μM NaH_2_PO_4_·2 H_2_O, 1.2 mM Na_2_SO_4_, 10.0 mM D-Glucose and 0.4 mM EDTA. ’Activated papain solution’ was prepared by mixing 8 U of papain (Worthington Biochemical, #LS003127) with 48 μl of ‘activator’ containing H_2_O with 1.1 μM EDTA, 5.5 mM L-cysteine and 0.07 mM 2-mercaptoethanol and incubating for 30 minutes at 37°C before diluting with 950 μl of 37°C ringer solution. Tissue pieces were incubated at 37°C in activated papain solution: 300 μl per organoid for 35 minutes; 500 μl per human retinal piece for 30 minutes. The papain digestion reaction was stopped by placing the tubes on ice and adding equal volumes of ‘stop solution’ containing Neurobasal A medium (Thermo Fisher Scientific, #10888022), 2 mM GlutaMAX (Thermo Fisher Scientific, #35050038), 10% FBS (Millipore, #es‒009‒b) and 20 U / ml DNase (Sigma, #D4263). Samples were centrifuged at 200 *g* and 4°C for 30 s and washed using Neurobasal A supplemented with 2 mM GlutaMAX and 10% FBS: 1 mL per organoid; 1.5 mL per retinal piece. A single cell suspension was generated by gently triturating 20 × with a 1,000-μl pipette tip in ice cold Neurobasal A (Thermo Fisher Scientific, #10888022), 2 mM GlutaMAX (Thermo Fisher Scientific, #35050038), 2% B27 supplement without vitamin A (GIBCO, #12587‒010), and 20 U / ml DNase (Sigma, #D4263) or until no large clumps were visible: 300 μl per organoid; 500 μl per human retinal piece. The cells were collected by centrifuging 5 minutes at 300 *g* and 4°C and resuspended in PBS containing 0.04% BSA (Thermo Fisher Scientific, #AM2616). The cell suspension was filtered to remove cell aggregates and particles with a diameter greater than 35 μm. Cells were mixed 1:1 with trypan blue (Thermo Fisher Scientific, #T10282) and counted using the Countess II cell counter (Thermo Fisher Scientific); cell viability was typically above 80%.

##### Single cell RNA-Sequencing

Cellular suspensions (8,000 cells per lane) were loaded on a 10x Genomics Chromium Single Cell instrument to generate single-cell Gel Beads in Emulsion (GEMs). Single-cell RNA-Seq libraries were prepared using GemCode Single Cell 3′ Gel Bead and Library Kit according to the manufacturer’s manual (version CG00052_SingleCell3′ReagentKitv2UserGuide_RevD, version CG000183_Rev_C for the version 3 kit). The Chromium Gene Expression version 2 (v2) kit was used for all samples except the ischemia time-course experiment for which the Chromium Gene Expression v3 kit was used. Reverse transcription of GEMs was performed in a Bio-Rad PTC-200 thermal cycler with a semi-skirted 96-well plate (Eppendorf, #0030 128.605): 53°C for 45 minutes, 85°C for 5 minutes, held at 4°C. After reverse transcription, the GEMs emulsion was broken and the single-stranded cDNA was cleaned up with DynaBeads MyOne Silane Beads (Life Technologies, #37002D). cDNA was amplified using a Bio-Rad PTC-200 thermal cycler with 0.2 mL 8-strip non-flex PCR tubes with flat caps (STARLAB, #I1402-3700): 98°C for 3 minutes, cycled 12 × : 98°C for 15 s, 67°C for the v2 kit and 63°C for the v3 kit for 20 s, and 72°C for 1 minutes; 72°C for 1 minutes, held at 4°C. Amplified cDNA product was cleaned up with the SPRIselect Reagent Kit (0.6 × SPRI; Beckman Coulter, #B23317). Indexed sequencing libraries were constructed using the reagents in the appropriate Chromium Single Cell 3′ library kit v2 (10x Genomics, #120237) or v3 (10x Genomics, #1000078), following these steps: (i) fragmentation, end repair and A-tailing, (ii) post fragmentation, end repair and A-tailing; double-sided size selection with SPRIselect Reagent Kit (0.6 × SPRI and 0.8 × SPRI), (iii) adaptor ligation, (iv) post-ligation cleanups with SPRIselect (0.8 × SPRI), (v) sample index PCR using the Chromium multiplex kit (10x Genomics, #120262), and (vi) post-sample index double-sided size selection with SPRIselect Reagent Kit (0.6 × SPRI and 0.8 × SPRI). The barcode sequencing libraries were measured using a Qubit 2.0 with a Qubit dsDNA HS assay kit (Invitrogen, #Q32854) and the quality of the libraries assessed on a 2100 Bioanalyzer (Agilent) using a high-sensitivity DNA kit (Agilent, #5067‒4626). Sequencing libraries were loaded at 10 – 12 pM on an Illumina HiSeq2500 with 2 × 50 paired-end kits using the following read length for the Chromium v2 kit: 26 cycles Read1, 8 cycles i7 Index, and 98 cycles Read2 and for the Chromium v3 kit: 28 cycles Read1, 8 cycles i7 index and 91 cycles Read2.

##### Microelectrode array recording and visual stimulation of human retinas

The neural retina and pigment epithelium / choroid, which had strong interconnections, were left attached to each other until a sample was recorded. In this manner, light responses could be recorded from samples of artificially perfused *ex vivo* human retina for up to 16 hours post mortem. Before recording, a small piece of retina (3 mm × 3 mm) was cut and the neural retina isolated from the pigment epithelium and choroid. The neural retina was placed onto a high-density microelectrode array and stabilized using a transparent permeabilized membrane (Polyester, 10-μm thickness, 200-μm hole size, 400-μm spacing). The high-density microelectrode array had the dimensions 3.85 × 2.1 mm and contained 26,400 platinum electrodes, of which 1,024 could have their electrical activity recorded simultaneously (Müller et al., 2015). A chamber surrounding the electrode array was perfused with Ames media (Sigma, #A1420) saturated with 95% O_2_ / 5% CO_2_ gas at 37°C. During recording, patterned light stimulation was delivered. Stimuli were generated by a DLP projector (K10, Acer) with optics modified to project onto the retinal surface. The projector was controlled by custom Python software developed by Z. Raics. The stimulus was a light step followed by a frequency chirp and an intensity sweep (Baden et al., 2016). Maximal light irradiance was 29.3 μW / mm^2^ in the plane of the retina and white light was generated using achromatic RGB triplets.

### Quantification and Statistical Analysis

#### Histology analysis

##### RNAscope analysis

On average we analyzed 847 individual regions of interest (ROIs) per tissue (n = 4 sections of human retina of periphery, fovea; n = two to six different retinal organoids from F49B7 and IMR90.4) throughout all retinal layers. Data was processed using tidyverse (v1.3.0) (Wickham et al., 2019). After background subtraction, averaged gray values of (ROIs) were *z*-normalized, separately for each test probe and on a per tissue basis, by

$$z=\;\frac{x-M}{k\;\cdot \;MAD}$$

where M is the median, MAD is the median absolute deviation and k is a constant scaling factor (*k* ≈1.4826) which allows the use of MAD as a consistent estimator of the standard deviation. ROIs that scored *z* > 4 for a given probe were considered positive for that probe. For a conservative estimation of the median and MAD, highly positive cells were masked out, i.e., the median and MAD were calculated only on values below an absolute threshold (*x* < 100 fluorescence units). Conclusions drawn from the study were not sensitive to the choice of threshold between 50 and 200 fluorescence units. This threshold was empirically derived from positive and negative experimental controls and uniformly used across all datasets, except in cases where it yielded a very low frequency of *RLBP1* positive ROIs (< 10%). In such cases, the threshold was iteratively lowered in steps of 10 fluorescence units until the aforementioned condition was met. The likelihood that the observed count of double positive ROIs for *RLBP1* and each target gene was non-random, conditioned on the respective marginal distributions, was computed using a Monte Carlo permutation test. *P*-values were estimated by approximating the tail of the distribution of permuted values on a generalized Generalized-Pareto distribution (Knijnenburg et al., 2009). *P*-values were adjusted for multiple comparisons by Bonferroni correction.

##### Locating cell types within retinal layers

Cells positive for a cell type marker were labeled with a circle as regions of interest (ROIs) to mark cell positions and the ROIs were projected onto the Hoechst channel. To quantify the localization of cell types within the human retina and retinal organoids the ImageJ macro “Points To Curve Distance Using Distance Map” was used. First, as a reference for the distance measurement, a line was drawn along the outside of the outer nuclear layer using the Hoechst channel. Second, the position of the nuclear layers was estimated using 10 marker points set along each of the inside edge of the outer nuclear layer, the outside edge of the inner nuclear layer, and the inside edge of the inner nuclear layer followed by setting marker points on the ROIs to measure the position of cells. The thickness of the retina was calculated by averaging the distance between the lines indicating the outer edge of the outer nuclear layer and inner edge of the inner nuclear layer; all other measurements were normalized to that distance.

#### Physiology analysis

##### Calcium activity imaging analysis

We motion-stabilized the recorded two-photon movies using rigid registration with a maximum (*x*,*y*) shift between frames of (6,6) (CaImAn, v1.8.2) (Giovannucci et al., 2019). To identify regions of interest (ROIs), we ran constrained non-negative matrix factorization on patches then refined the results by repeating the process on the components accepted by the first run. Components passed our quality control standards if their signal to noise ratio was above 0.7 and their space correlation was above 0.5. Since many cell types in the retina do not fire action potentials, we calculated signal to noise ratio as the ratio of signal below 1 Hz and signal above 1 Hz (GCaMP6s half decay time for action potentials is 455 ± 40 ms (Dana et al., 2019)). Multisession registration was used to combine ROIs across experiments, and spatial masks of sources identified by CaImAn were used to calculate the dF / F_0_ on the motion-stabilized movies. Traces were high-pass filtered to remove drift using a 2-pole Butterworth filter applied in both directions to prevent phase shift (SciPy, v1.4.1) (Virtanen et al., 2020).

##### Electrophysiology analysis

The raw electrical activity on the array was spike sorted using an automatic spike sorter dedicated to high-density microelectrode arrays to identify sets of action potentials arising from the same cell. Because individual spikes were detected on multiple electrodes, the sorter could cluster them using the combined information of the shapes and spatial extent of their waveforms (Diggelmann et al., 2018). Retinas were stimulated with a temporally repeating pattern of spatially uniform white light. Each repetition of the stimulus was considered a trial $Q_{k}$ and an entire experiment Q consisted of $n_{\text{trial}}$ trials. Spikes from each 30 s trial were binned into $n_{\text{bin}}=120$ bins of width 250 ms to create the matrix ρ with dimensions $n_{\text{bin}}\times n_{\text{trial}}$ whose elements contain the spike counts of each bin in each trial.

*Test for light-responsivity*. Light responsivity was assessed by testing for reproducibility of cells’ peristimulus activity across trials. For electrode arrays the activity was spike counts and for calcium imaging it was dF / F_0_. We describe the test for electrode array data, but the process was the same for calcium imaging except where otherwise noted. Spike counts were correlated between all possible combinations of pairs of trials at once. For example, when $n_{\text{trial}}=3$, the number of possible combinations of pairs of trials is $\left(\begin{array}{c} n_{\text{trial}}\\ 2 \end{array}\right)=3$. These three pairs are ⟨(1,2),(1,3),(2,3)⟩. The vectors containing the binned spike counts for these three pairs of trials would then be

$$v_{1}=\left(\rho_{1\cdot },\rho_{1\cdot },\rho_{2\cdot }\right)$$

$$v_{2}=\left(\rho_{2\cdot },\rho_{3\cdot },\rho_{3\cdot }\right)$$

with $\rho_{i\cdot }=\left(\rho_{i1},\rho_{i2},\ldots ,\rho_{in_{bin}}\right)$ the binned spike counts for each trial i.

For electrode arrays, cells were evaluated if their average firing rate exceeded 1/3 Hz (n = 4,073 of 4,343 cells) and considered light responsive if their Pearson correlation coefficient $R_{\text{P}}\left(v_{1},v_{2}\right)$ was positive and significant at P < 0.01 (n = 3350 of 4073 cells). The reported peak firing rate of a cell was the inverse of the average of the 5 shortest inter-spike intervals across the experiment. For calcium imaging, there was no minimum activity rate but cells had to be detected in the experiment and pass quality control to be considered for light responsivity.

For electrode array, peristimulus activity traces of light responsive cells were clustered by the Infomap method. First, K-nearest neighbors were approximated (Annoy v1.15.2, Spotify) using 200 trees and K=60. Second, the graph was clustered by unsupervised Infomap (Rosvall and Bergstrom, 2008) using the python-igraph library (Csardi and Nepusz, 2005). For visualization, light responses were embedded using an adaptation of the scVis algorithm (Ding et al., 2018) for electrophysiology data. Briefly, the t-SNE objective function was implemented inside a variational autoencoder (InfoVAE) (Zhao et al., 2017) implemented in Keras, using the TensorFlow backend, and trained with a perplexity of 30.

Clusters of cells with at least five distinct response types were isolated (Figures 4C and 4D). Cells of the first and second cluster showed responses at light onset (ON cells), with an increase in firing rate that was either sustained (cluster 1) or transient (cluster 2). The third cluster had ON-OFF cells that were transient, spiking briefly at both light onset and offset. The responses of the fourth and fifth clusters were the converse of clusters one and two, spiking at light offset (OFF cells) in either a transient (cluster four) or sustained (cluster five) manner.

#### Single-cell RNA sequencing analysis

##### Read alignment and expression count table generation

The Cell Ranger package (v2.0 or v3.1, 10x Genomics) was used to extract unique molecular identifiers, cell barcodes, and genomic reads from the sequencing results of 10x Chromium experiments. Reads were aligned against the annotated human genome (GRCh38, GENCODE v27), including both protein coding and non-coding transcripts. Reads with the same cell barcode and unique molecular identifier were collapsed to a unique transcript. In order to discard potentially unhealthy or damaged cells, the built-in cell filtering step of Cell Ranger was followed by further cell filtering using as criteria the number of detected transcripts and the fraction of mitochondrial reads. For both criteria, a LOESS fit was performed on the corresponding feature’s rank-size distribution; the slope of the fitted curve was determined at steps of relative width 1 / 10,000. The threshold was then set at inflection points past the 5^th^ (number of detected genes) or 95^th^ (fraction of mitochondrial reads) percentiles of the distributions. On average, an additional 2.5% of the cells were removed at this step. Transcripts from mitochondrial- and ribosomal-protein coding genes, which are typically very highly expressed and highly dispersed, irrespective of biological identity, were also discarded during embedding and clustering.

Two random variables were defined on the sample space of unique transcripts: the ‘cellular identity’ and ‘genetic identity’. Letting κ be the total number of unique transcripts, the random variable cellular identity (C) groups transcripts with matching cell barcodes into mutually disjoint subsets $C_{1},\;C_{2},\;\ldots ,\;C_{n_{\text{cell}}}$ where $n_{\text{cell}}$ is the number of single cells and the cardinality $\left|C_{i}\right|$ is the number of transcripts in cell $C_{i}$ such that $\kappa =\sum_{i=1}^{n_{\text{cell}}}\left|C_{i}\right|$. Formally, the values of C are $1,\;2,\;\ldots ,\;n_{\text{cell}}$ corresponding to the subsets $C_{1},\;C_{2},\;\ldots ,\;C_{n_{\text{cell}}}.$ Similarly, the random variable genetic identity (G) groups transcripts together that belong to the same gene. The values of G are the mutually disjoint subsets $G_{1},\;G_{2},\;\ldots ,\;G_{n_{\text{gene}}}$ where $n_{\text{gene}}$ is the total number of genes in the annotation. $G_{j}$, therefore, represents a particular gene and contains each transcript of this gene. If the cardinality $\left|G_{j}\right|$ is the total number of transcripts in $G_{j}$ then $\kappa =\sum_{j=1}^{n_{\text{gene}}}\left|G_{j}\right|$. The contingency table between C and G is the count matrix M whose elements $M_{ij}$ are the number of transcripts observed in cell $C_{i}$ and gene $G_{j}$.

##### Embedding transcriptomes into maps with scVis

Let $\mu_{G_{j}}$ and $\sigma_{G_{j}}$ denote the mean and standard deviation of the transcript counts for gene $G_{j}$ across all cells and α=0.8 be a trend adjustment that was empirically estimated on held-out data. The trend-adjusted log coefficient of variation was

$$CoV_{G_{j}}=log_{2}\left(1+\sigma_{G_{j}}\right)-\alpha \;log_{2}\left(1+\mu_{G_{j}}\right)$$

Adding 1 before taking the logarithm preserved matrix sparsity, kept values positive, and flattened the gradient for the smallest expression values. The 1,000 genes with the highest $CoV_{G_{j}}$ were retained for dimensionality reduction. Gene selection was performed separately for organoids and adult retina.

The average number of transcripts per cell in the sample was

$$\bar{\kappa }=\frac{\kappa }{n_{\text{cell}}}$$

Let $M_{i\cdot }=\left(M_{i1},M_{i2},\ldots ,M_{in_{gene}}\right)$ denote the vector of per-gene transcript counts for cell $C_{i}$ and recall that $\left|C_{i}\right|$ is the total transcript count for cell $C_{i}$. We define the scaled normalized expression vector as

$${\hat{M}}_{i\cdot }=\frac{\bar{\kappa }}{\left|C_{i}\right|}M_{i\cdot }$$

Because the expression values were non-negative $\left|{\hat{M}}_{ij}\right|={\hat{M}}_{ij}$ and the normalized expression’s $L_{1}$ norm $\left\|{\hat{M}}_{i\cdot }\right\|$ is equal to its sum.

$$\left\|{\hat{M}}_{i\cdot }\right\|=\sum_{j=1}^{n_{\text{gene}}}\left|{\hat{M}}_{ij}\right|=\sum_{j=1}^{n_{\text{gene}}}{\hat{M}}_{ij}$$

After normalizing, the sum of the expression in each cell was the same $\left\|{\hat{M}}_{1\cdot }\right\|=\left\|{\hat{M}}_{2\cdot }\right\|=\left\|{\hat{M}}_{n_{\text{cell}}\cdot }\right\|$ and equal to $\bar{\kappa }$, the average transcript count per cell before normalizing. The normalized expression values were then converted to a logarithmic scale, $log_{2}\left(1+{\hat{M}}_{i\cdot }\right)$, and incremental principal component analysis was performed to estimate scores for the top 100 principal components.

Before the final dimensionality reduction step, cells with very few transcripts were removed, in response to observations that their additional noise negatively influenced the learned embedding. Cells were split into a high- and a low-expression pool based on $\left|C_{i}\right|$. Thresholds for high expression were – adult: $\left|C_{i}\right|>800$, and organoid: $\left|C_{i}\right|>400$. The resulting percentage of cells in the low-expression category were – peripheral: 49.4%, foveal: 3.2%, and organoid: 0.5%. The majority of cells removed in the peripheral retinal samples were rods (estimated 77.7% of the 49.4%), which are highly-represented in the peripheral dataset and can dominate the analysis if not depleted (Macosko et al., 2015).

For organoids of all ages, the high-expression pool was embedded into a 2-dimensional matrix using the scVis method (Ding et al., 2018). Parameters supplied to scVis were – Adam optimizer, learning rate of 0.01, batch size of 512, $L_{2}$ regularization strength of 0.001, perplexity of 10, and an ELU activation layer. Library normalization and transcript embedding were performed in Python 2.7 (NumPy v1.14.3, SciPy v0.18.1, scikit-learn v0.19.1, TensorFlow v1.9.0) (Pedregosa et al., 2012; van der Walt et al., 2011).

##### Heatmaps of organoid single cell data

Let S represent the $n_{\text{cell}}\times 2$ dimensional scVis map of organoid cell transcriptomes. The estimator ${\hat{f}}_{h}$ is a 2D Gaussian kernel density estimator used to calculate $\hat{S}$, the estimated density of S. The kernel bandwidth h was estimated by Scott’s rule (Scott, 2015).

$$\hat{S}=\;{\hat{f}}_{h}\left(S\right)$$

The elements of $\hat{S}$ are ${\hat{S}}_{xy}$ and its dimensionality was 341 × 341, evaluated between −17.0 and +17.0 with a step size of 0.1. For visualization purposes, an isodensity line was drawn at $\hat{S}=2\times 10^{-4}$. A subset of organoid cells $C^{'}\subseteq C$ was selected to analyze organoids from different ages, batches, etc. Letting $n_{\text{cell}}^{'}$ represent the number of cells in C’, the scVis map for these points is the matrix $S^{'}$ with dimensions $n_{\text{cell}}^{'}\times 2$. Holding h constant, the density of the subset was estimated as $\hat{S^{'}}={\hat{f}}_{h}\left(S\text{'}\right)$ whose elements ${\hat{S'}}_{xy}$ were evaluated at the same 341 × 341 locations. The relative density of the subset of cells across the map is

$$D\left(S_{xy}^{\prime }\right)=\frac{{\hat{S'}}_{xy}}{{\hat{S}}_{xy}+\varepsilon }$$

where the constant ε=0.01 suppresses noise in regions with low density.

We used the weighted kernel density estimator ${\hat{g}}_{h}\left(S,w\right)$ to find $\Psi \left(G_{j}\right)$, the estimated location for transcripts of gene $G_{j}$ within the scVis map S (Figure S2). In addition to the arguments accepted by ${\hat{f}}_{h}$, the estimator ${\hat{g}}_{h}$ accepts the weight vector w which was set to the transcript count for gene $G_{j}$ across all cells, $w=M_{\cdot j}$. The bandwidth h was held constant.

$$\Psi \left(G_{j}\right)={\hat{g}}_{h}\left(S,\;M_{\cdot j}\right)$$

If the elements of $\Psi \left(G_{j}\right)$ are $\Psi_{\text{xy}}\left(G_{j}\right)$, the relative density of transcripts across the map is

$$D\left(S_{xy},\;G_{j}\right)=\frac{\Psi_{\text{xy}}\left(G_{j}\right)}{{\hat{S}}_{xy}+\varepsilon }$$

##### Stabilization of developing transcriptomes

Organoid transcriptomes at different ages were compared in the context of their scVis map kernel-density estimates. The 2-dimensional density estimates for a subset of cells $\hat{S^{'}}$ were $L_{1}$ normalized by the total estimated kernel density $\left\|\hat{S^{'}}\right\|$ to create the probability mass function $Q\left(S^{'}\right)=\left(\hat{S^{'}}/\left\|\hat{S^{'}}\right\|\right)$. Two cell subsets, $S_{b}^{\;'}$ and $S_{d}^{\;'}$, were compared by calculating their respective probability mass functions, $Q\left(S_{b}^{\;'}\right)$ and $Q\left(S_{d}^{\;'}\right)$, and calculating the Jensen-Shannon divergence between the two distributions. Briefly, if the average of their two probability mass functions is $M=\left(\left(Q\left(S_{b}^{\;'}\right)+Q\left(S_{d}^{\;'}\right)\right)/2\right)$ their Jensen-Shannon divergence is $D_{\text{JS}}=\left(D_{\text{KL}}\left(Q\left(S_{b}^{\;'}\right)\left|\right|M\right)/2\right)+\left(D_{\text{KL}}\left(Q\left(S_{d}^{\;'}\right)\left|\right|M\right)/2\right)$ where $D_{\text{KL}}$ is the Kullback-Leibler divergence.

##### Comparing the rate of organoid and human development

We used an available developing retina transcriptome from bulk (i.e., not single-cell) samples of human retina (Hoshino et al., 2017). Replicates were averaged and, to keep the approach region-agnostic, transcriptomes from specific retinal regions (e.g., peripheral retina) were excluded. Pseudo-bulk gene expression vectors were generated from our single cell data by using $\left|G_{j}^{C^{'}}\right|$, the number of unique transcripts observed for gene $G_{j}$ within a subset of cells C’ (here, the subset of cells from an individual organoid). To focus subsequent analysis steps on genes likely to be modulated during development, we selected a gene $G_{j}$ for analysis if the median-normalized range, $dnr_{G_{j}}$, of its vector of bulk / pseudo-bulk expression across tissues from different ages $B_{G_{j}}$ was greater than 0.5

$${\text{dnr}}_{G_{j}}={\text{ log}}_{2}\left(1+\max\left(B_{G_{j}}\right)\right)-\log_{2}\left(1+\min\left(B_{G_{j}}\right)\right)-0.25\;\log_{2}\left(1+\text{median}\left(B_{G_{j}}\right)\right)$$

The number of genes thus selected was – organoids: 613; developing retina: 212. Because not all cell classes / types were present in each tissue (e.g., organoids lacked vasculature and immune cells, and the retinas lacked pigment epithelium) the intersection of the two gene sets, containing 93 genes, was used for both datasets.

A correlation matrix was constructed by determining the Spearman correlation coefficients, $R_{\text{S}}$, of each individual retina / organoid to the others. To predict retina-equivalent ages of organoid transcriptomes we used ordinary least-squares linear regression. Since genes outnumber the available retinas, we combatted overfitting by regressing against a transcriptome’s correlations to the retina transcriptomes. First, the model was trained to predict retinal ages from retina-retina correlations. Some correlations along the diagonal reaching 0.8, suggestive of co-modulation in gene expression during development (Figure 3H). The training data was the set of vectors of $R_{\text{S}}$ between each retinal transcriptome and all retinal transcriptomes, and the target values were the retinal ages. To evaluate the model, leave-one-out cross validation was applied; the age of each retinal transcriptome was predicted by a model trained on the other retinal transcriptomes. Second, to quantify developmental transcriptomic changes, we generated two self-to-self correlation matrices, one for retina (Figure 3I) and one for organoids (Figure 3J). In these matrices, gene correlations peaked for adjacent sample ages and extended for many weeks in either direction suggesting that it is possible to predict the retina-equivalent age of developing organoids. The trained model was applied to the retina-organoid data; the input for an organoid was the vector of $R_{\text{S}}$between its transcriptome and each retinal transcriptome. Because the pattern of positive correlations extended for weeks in either direction, the model predicted a retinal-equivalent age outside of the range of retina ages for some organoid transcriptomes.

##### Multiplet removal

Multiplets occur when one cell barcode shares transcripts from multiple single cells due to incomplete dissociation or well-dissociated single cells being placed in the same droplet. Transcriptomes likely to be multiplets were first identified by the presence of genetic markers for two or more cell classes or sets of classes. The sets of markers were – rods: *GNGT1*, *NRL*, *PDE6G*, *RHO*; cones: *ARR3*, *CNGA3*, *OPN1LW*, *OPN1MW*, *OPN1SW*, *PDE6H*; horizontal cells: *LHX1*, *LNP1*, *ONECUT1*, *VAT1L*; bipolar cells: *GRIK1*, *IRX6*, *LRTM1*, *PCP2*, *PRKCA*, *TRPM1*, *VSX1*, *VSX2*; amacrine cells: *GAD1*, *SLC6A9*, *TFAP2A*, *TFAP2B*; ganglion cells: *POU4F2*, *NEFL*, *NEFM*, *RBPMS*, *SLC17A6*, *SNCG*, *THY1*; pigmented cells: *BEST1*, *MITF*, *MLANA*, *TJP1*, *RPE65*; glial cells: *CRABP1*, *GFAP*, *GLUL*; endothelial cells, mural cells and fibroblasts: *ACTA2*, *COL1A2*, *EGFL7*, *PDGFRB*, *PROCR*, *VWF*; immune cells: *AIF1*, *CD2*, *CD48*, *CX3CR1*, *HBB*, *IL32*, *JCHAIN*, *LST1*.

For a cell $C_{i}$ and a set of marker genes G' containing gene indices J, a score was created by summing transcript counts, $score_{C_{i}}\left(G^{'}\right)=\sum_{j\in J}M_{ij}$. If marker specificity for the target cell class was perfect and background RNA was absent, the score distribution would contain a mode with positive expression (cells of the class) and a non-overlapping mode at zero (cells not of the class). In practice, the specificity is imperfect and the distribution more complicated. The threshold score for separating cells of the class from cells not of the class were empirically estimated for each set of marker genes independently. Cells with a $score_{C_{i}}$ above threshold for two or more marker sets were flagged as multiplets. Additional doublets were then removed using Scrublet (Wolock et al., 2019). The transcriptomes of cells removed as multiplets were commonly present in low density regions of scVis maps, especially in the space between pairs of populous cell types (e.g., between rods and rod bipolar cells, rods and Müller cells) which is a rational placement for doublets. Because $score_{C_{i}}$ relies on a small number of genes and transcripts are prone to dropout it is susceptible to false negatives. Therefore, cells were also labeled multiplets if they shared a cluster with a high percentage of multiplets: > 30% for adult retina; > 15% for organoid. The total percentages of cells removed as multiplets were: peripheral: 5.7%; foveal: 6.1%; developing organoids: 1.0%; developed organoid: 5.0%.

##### Infomap clustering and cluster merging

We constructed a graph from the scores of cells for the first 100 principal components using K nearest-neighbor search with K=50 (LargeVis v0.2.1) (Tang et al., 2016). Clusters of cells with similar transcriptomes were identified by applying Infomap clustering (Rosvall and Bergstrom, 2008) to the graph (igraph v1.1.2). For each transcript, the random variable ‘cluster identity’ T was generated by mapping the cell identity C to its corresponding Infomap cluster $T_{k}$. The values of T are the mutually disjoint subsets $T_{1},\;T_{2},\ldots ,\;T_{n_{\text{clust}}}$ where $n_{\text{clust}}$ is the total number of clusters. $T_{k}$ represents a particular cluster of cells and contains the transcripts of each cell in that cluster. If $\left|T_{k}\right|$ is the number of transcripts in cluster $T_{k}$ then $\kappa =\sum_{k=1}^{n_{\text{clust}}}\left|T_{k}\right|$. Similarly, if $m_{T_{k}}$ represents the number of cells in cluster $T_{k}$ then $n_{\text{cell}}=\sum_{k=1}^{n_{\text{clust}}}m_{T_{k}}$. The contingency table between T and G is the count matrix N whose elements $N_{kj}$ are the number of transcripts observed in cluster $T_{k}$ and gene $G_{j}$. The normalized expression matrix $\hat{N}$ whose elements ${\hat{N}}_{kj}$ are the summed normalized expression values in cluster $T_{k}$ and gene $G_{j}$ generated by summing the normalized expression vectors ${\hat{M}}_{i\cdot }$ for each cell $C_{i}$ in cluster $T_{k}$. These clusters contain groups of cells with different transcriptomic ‘cell types’.

To assess cluster quality of each dataset, we iteratively repeated the clustering 50 times. In each iteration, 85% of the high-expression cells were sub-sampled and the graph construction and Infomap clustering were repeated. Measures of cluster purity and stability during resampling were the same as those defined in similar studies to improve comparability (Shekhar et al., 2016). Cluster purity and stability were both high in the peripheral retina, foveal retina, and in developed organoids (peripheral retina: mean / 5^th^ percentile purity = 0.97 / 0.87, stability = 0.96 / 0.88; foveal retina: purity = 0.96 / 0.78, stability = 0.95 / 0.81; developed F49B7 organoids: purity = 0.94 / 0.79, stability = 0.93 / 0.80) (Figure S2).

Since the presence of batch effects in the scVis map indicated the potential for over-clustering, highly similar clusters were merged iteratively: pairs of clusters with a Pearson correlation coefficient of cluster-averaged gene expression $R_{\text{P}}$ were merged if $R_{\text{P}}>\;\theta_{\text{merge}}$; $\theta_{\text{merge}}$ was initialized to 0.99 and clusters were iteratively combined for $R_{\text{P}}>\;0.90$. High-expression cells were selected from the count matrix, and genes were selected if their mean-normalized log-range, $mnr_{G_{j}}$, was greater than the empirically estimated threshold 4.0.

$$mnr_{G_{j}}=\;log_{2}\left(1+\text{max}\left(M_{\cdot j}\right)-min\left(M_{\cdot j}\right)\right)-\alpha \;log_{2}\left(1+\mu_{G_{j}}\right)$$

where $\text{max}\left(M_{\cdot j}\right)$ and $min\left(M_{\cdot j}\right)$ were the highest and lowest expression counts for gene $G_{j}$ observed in the cell sample and α=0.8 was a trend adjustment empirically estimated on held-out data. The number of genes thus selected were – peripheral: 632, foveal: 1273, and organoid: 705. Mergers were resolved in an inclusive fashion (e.g., if cluster 2 met the merger criterion with clusters 1 and 3, all three clusters would be merged together even if clusters 1 and 3 did not meet the merger criterion with each other).

##### Subclustering

Since the retina has a hierarchical cell type organization, with nested groups of related cells, the Infomap clusters identified above were manually annotated based on known markers and placed into 10 mutually exclusive groups of cell types (‘cell classes’) and subclustered. The cell classes were: rods, cones, horizontal cells, bipolar cells, amacrine cells, ganglion cells, macroglia, pigmented cells, vascular / fibroblast cells, and immune cells. Annotation was performed independently for the peripheral retina, foveal retina, and developed organoids.

During subclustering the SCANPY framework (v1.4.6) (Wolf et al., 2018) was used to load, process, and store data. For each cell class, the top 500 overdispersed genes were identified for each of fovea and periphery. The union of these two gene sets was the representation used during subclustering. Transcriptomes from the peripheral retina and foveal retina were concatenated to form an adult retinal count matrix. Adult retina and developed organoid count matrices were each centered and scaled to unit variance, with values clipped at 10. The resulting peripheral retina, foveal retina, and developed organoid transcriptomes were integrated using the mnnpy implementation of MNN correction (Haghverdi et al., 2018). The combined and adjusted count matrix was converted to a nearest neighbor graph (ANNOY, v1.15.2, Spotify) using Euclidean distances. Because MNN correction operates using Euclidean distances, performance was best when the same distance metric was used to create graphs from corrected data. For each class Infomap clustering was performed on this graph. Cells were embedded with UMAP (McInnes et al., 2018) (Figure S2). The integrated count matrix was not used in any subsequent analyses.

Infomap clusters (‘cell types’) were aggregated from each cell class to form the final cell type atlas. Cell types were annotated manually according to known markers. We excluded two clusters containing cells miscategorized during the initial Infomap clustering from further analysis. For quality control reasons, if a cell type contained less than 10 cells within the peripheral retina, foveal retina, or developed organoid it was considered to not be present for the purpose of reporting statistics and gene expression patterns. If peripheral retina and foveal retina were combined into ‘retina’, the threshold was 10 cells in aggregate.

The maps illustrating cells from all classes (Figures 5B–5D) were created by semi-supervised UMAP embedding. Cell classes, but not cell types, were provided to UMAP to help constrain the fit to favor nearby placement of cells within the same class (Figure S2).

##### Regional character of cell type composition

Cell type composition was compared by Spearman correlation, *R*_s_, and a χ^2^ test of the categorical distribution for each cell type between the peripheral and the foveal retina (significantly different at p = 2.2 × 10^−308^, χ^2^ test) (Figure S4), developed organoids and the peripheral retina (*R*_s_ = 0.61, p = 2.9 × 10^−5^), and between developed organoids and the foveal retina (*R*_s_ = 0.13, p = 1.00). When ganglion cells were excluded from the comparison, the correlation with the periphery remained but was lower (periphery: *R*_s_ = 0.60, p = 7.9 × 10^−5^; fovea: *R*_s_ = 0.28, p = 0.29), suggesting that the low number of ganglion cells in both developed organoids and peripheral retina does not fully account for their high correlation. All *P*-values were Bonferroni adjusted.

##### Regional transcriptomic character of cell classes

The following process was repeated for different cell classes or types. In cases, multiple related cell types were combined and analyzed as a single cluster. Recall from above that T is the cluster identity of transcripts, and the subset of transcripts belonging to a cluster is $T_{k}$. The number of cells in cluster $T_{k}$ is $m_{T_{k}}$ and $\hat{N}$ is the expression matrix of size $n_{\text{clust}}$ × $n_{\text{gene}}$ whose elements ${\hat{N}}_{kj}$ are the sum of the normalized expression values in cells of cluster $T_{k}$ for gene $G_{j}$. Since peripheral and foveal datasets were processed separately, superscripts fov and per are employed to distinguish between variables referring to the two datasets. Cells from foveal retina and developed organoid were scaled to have the same library size as the peripheral retina. To simplify the notation, the same index k is used to refer to the cluster in both datasets, as if the cluster indices were ordered by cell class. For example, the notation for cluster $T_{k}$ in the peripheral and foveal retina are $T_{\;k}^{\text{per}}$ and $T_{\;k}^{\text{fov}}$ and for normalized expression matrix $\hat{N}$ they are ${\hat{N}}^{\text{per}}$ and ${\hat{N}}^{\text{fov}}$. The cell-averaged expression of gene $G_{j}$ within cluster $T_{k}$ in the foveal retina was

$$\mu_{T_{k}^{\text{fov}}G_{j}}=\frac{{\hat{N}}_{kj}^{\text{fov}}}{m_{T_{k}^{\text{fov}}}}$$

And the cell-averaged mean expression for the two regions is

$$\mu_{T_{k}G_{j}}=\frac{{\hat{N}}_{kj}^{\text{per}}+{\hat{N}}_{kj}^{\text{fov}}}{m_{T_{k}^{\text{per}}}+m_{T_{k}^{\text{fov}}}}$$

In addition to considering $\mu_{T_{k}G_{j}}$, the average transcript counts within cluster $T_{k}$, we also considered $\mu_{T_{o}G_{j}}$, the average transcript counts in all clusters other than cluster $T_{k}$

$$\mu_{T_{o}G_{j}}=\frac{{\hat{N}}_{oj}^{\text{per}}+{\hat{N}}_{oj}^{\text{fov}}}{m_{T_{o}^{\text{per}}}+m_{T_{o}^{\text{fov}}}}$$

50% of the single cells were selected at random and held-out for later cross-validation. On the remaining transcriptomes, genes were classified as overexpressed if: $log_{10}\left(\frac{\mu_{T_{k}G_{j}}}{\mu_{T_{o}G_{j}}}\right)>\;0.1$, $\mu_{T_{k}G_{j}}>1$ and $\mu_{T_{o}G_{j}}<10$. To limit the potential influence of low levels of background transcripts from other clusters, further analysis of the cluster’s transcriptome was restricted to these overexpressed genes.

For gene $G_{j}$ in the context of cluster $T_{k}$, the index of regional specificity was

$$specificity_{T_{k}G_{j}}=\frac{\mu_{T_{k}^{\text{fov}}G_{j}}-\mu_{T_{k}^{\text{per}}G_{j}}}{\mu_{T_{k}^{\text{fov}}G_{j}}+\mu_{T_{k}^{\text{per}}G_{j}}}$$

The significance of regional expression differences for gene $G_{j}$ was evaluated by the Mann-Whitney U test with continuity correction on the normalized expression vectors ${\hat{M}}_{\cdot j}^{\text{fov}}$ and ${\hat{M}}_{\cdot j}^{\text{per}}$. Since *P*-values falling below 1.8 × 10^−307^ underflowed to zero, they were visualized by assigning a random value between 10^−350^ and 10^−400^.

A classifier was trained by least-squares regression to predict the region-of-origin for single cells of the cluster based on their transcriptomes. The combined foveal and peripheral datasets were standardized and each set was shuffled and trimmed so that the number of cells per region, m, was equal and at most 1,000. The peripheral and foveal transcriptomes were combined to create a 2m×d set of observations to train the model, where d is the number of overexpressed genes for that cluster. The model output to be predicted was the length 2m vector containing the target regional labels from paired observations; the label for peripheral retina was mapped to −1, foveal retina to +1. For cell $C_{i}$, the model for the regional classifier was

$$r_{C_{i}}=\tanh\left(\frac{1}{n_{\text{gene}}}\sum_{j=1}^{n_{\text{gene}}}\beta_{G_{j}}M_{ij}\right)$$

The model coefficients β are an $n_{\text{gene}}$ length vector whose element $\beta_{G_{j}}\;$is initialized to 10% of the regional specificity index for gene $G_{j}$. The optimization cost function was least-squares with an $L_{2}$ regularization weight of 1 × 10^−3^. After fitting the model, held-out data was used for cross-validation. Let $r_{C_{i}}^{\text{cv}}$ be the regional classifier score for cell $C_{i}$ from the cross validation with held-out data. Let $y_{C_{i}}$ be the target regional labels of the cross-validation and $n_{\text{cv}}$ be the number of cells in the cross-validation. The classifier’s coefficient of determination, $R^{2}$, was then

$$R^{2}=1\;–\frac{\sum_{i=1}^{n_{\text{cv}}}{\left(y_{C_{i}}-r_{C_{i}}^{\text{cv}}\right)}^{2}}{\sum_{i=1}^{n_{\text{cv}}}{\left(y_{C_{i}}-\bar{y}\right)}^{2}}$$

where

$$\bar{y}=\frac{1}{n_{\text{cv}}}\sum_{i=1}^{n_{\text{cv}}}y_{C_{i}}$$

The performance of the classifier was quantified using the coefficient of determination (R^2^), which takes values between zero and one, with higher values for more accurate classifiers and a value of one when all cells are correctly labeled as peripheral or foveal. The classifiers all had coefficients of determination above 0.25, with the largest differences in gene expression between periphery and fovea are in Müller and pigment epithelial cells. The main periphery-specific genes in Müller cells included *ATP1A2*, *COL2A1*, *KCNK1*, and *MT3* and in pigment epithelial cells *TFPI2*, *FXYD3*, and *GAP43*. The main fovea-specific genes in Müller cells included *FAM237B*, *RHCG*, *PMP2*, *CYP26A1*, and *EFEMP1* and in pigment epithelial cells *WFDC1*, *CHI3L1*, and *CHN1*. The periphery-specific gene *COL2A1* is associated with Stickler syndrome, and the fovea-specific gene *EFEMP1* is associated with Doyne honeycomb macular dystrophy. *In situ* hybridization was used to evaluate predicted peripheral markers *ATP1A2* and *COL2A1* and foveal markers *FAM237B* and *RHCG* against the Müller cell marker *RLBP1* (Figure S4). In all cases the regional specificity of the markers was as predicted by the transcriptome and supported the classifier results.

In organoids, individual cells could have transcriptomes with peripheral or foveal characteristics. The regional identity of developed organoid cells was estimated using the previously described model (classifier) that successfully identified the regional origin of adult retinal cell types. For each cell class that was present in organoids, single cell transcriptomes underwent the same pre-processing and their $r_{C_{i}}$ was calculated to predict their regional character. Organoids cells were defined as ‘overlapping’ the adult cell distribution if they were below the 95^th^ percentile of peripheral cells or above the 5^th^ percentile of foveal cells (Figure S4). If a mutually overlapping interval was present, cells in its range were excluded. The model classified a cell class as “predominantly peripheral” if > 50% had scores overlapping the peripheral cell distribution, < 10% had scores overlapping the foveal cell distribution, and the observed distribution was significant by χ^2^ test at p < 0.05; the converse percentages were “predominantly foveal” (Table S3).

The classifier’s prediction that Müller cells in organoids were peripheral in character was evaluated by a second method. *In situ* hybridization confirmed the that organoids (Figure S4) expressed the peripheral markers *ATP1A2* (F49B7, p = 1.4 × 10^−30^ by Monte Carlo permutation test with Bonferroni correction; IMR90.4, p = 1.5 × 10^−20^) and *COL2A1* (F49B7, p = 1.1 × 10^−33^; IMR90.4, p = 2.7 × 10^−26^) but not the foveal markers *FAM237B* (F49B7, p = 1.0; IMR90.4, p = 1.0) and *RHCG* (F49B7, p = 1.0; IMR90.4, p = 1.0) within Müller cells (*RLBP1* positive).

##### Closeness of organoids to adult cell classes

For a peripheral retina cell type, we identified the top 20 marker genes for each cell type and performed principal component analysis (PCA) on the expression values of these genes in cells of the chosen retinal cell type as well as in cells of organoids from week six to 38, regardless of their type. We examined the first two PCA components (PCA map) for each cell at different time points during organoid development. The procedure below was applied to a cell class. Genes differentially expressed for a cell class were defined by the metric

$$dge_{T_{k}G_{j}}=\log_{10}\left(\mu_{T_{k}G_{j}}+\varepsilon \right)-\log_{10}\left(\mu_{T_{o}G_{j}}+\varepsilon \right)$$

where ε=0.5 was a constant. Principal component analysis was performed using the 20 genes with the highest $dge_{T_{k}G_{j}}$ as the dimensions, and a combination of (i) all peripheral retinal cells from the cell class and (ii) a number of organoid cells, equal to the number of peripheral retinal cells, subsampled without respect to organoid age or cell class. The resulting principal components were used to project the remaining organoid cells.

As an example, at week six, the characteristics of organoid cells were far away from those of cones of the adult peripheral retina (Figure S4). With time, a group of organoid cells moved closer on the PCA map to the cloud of adult cones and by week 38 developed organoid cells intermingled with adult cones in terms of gene expression.

To quantify this phenomenon, we defined organoid cells as being ‘close’ to an adult cell type if their distance from the adult distribution was below three (three SD) or five standard deviations (five SD) in the PCA map. The distribution of the first two principal components (PC1, PC2) of adult cells was fit using the graphical lasso algorithm. The Mahalanobis distance of each organoid cell to this distribution was then calculated to assess how ‘close’ organoid cells were to the adult cell distribution within the space of the top cell class-specifying genes. This comparison was restricted to individual cell types such as rods, cones, Müller cells, and pigment epithelial cells. We further defined the ‘closeness’ of a particular cell type as the percentage of cells of that type in the developed organoid that were close to the adult peripheral cluster. The closeness values at week 38 were, for three SD and five SD respectively: rods, 0.4% and 0.9%; cones, 52.9% and 70.2%; Müller cells, 38.2% and 59.9%; pigment epithelial cells, 0.0% and 0.0%.

##### Distance between organoid and adult peripheral gene expression

The following was repeated for every cell type that was present (at least 10 cells from the cell type in each tissue) in the adult peripheral retina and developed organoid. The sample mean gene expression profile was calculated for the adult, and the Euclidean (L_2_) distance from every individual cell’s transcriptome to the type mean transcriptome was calculated. The mean distance was calculated for this distribution and its standard deviation was estimated using the median absolute deviation. The distance from every organoid cell to the adult type mean transcriptome was then calculated and standardized into a *z*-score by subtracting the mean distance from the adult and dividing by the adult distribution’s standard deviation.

##### Mapping genes to cell classes

The distribution of genes across clusters was determined by taking $\mu_{T_{k}G_{j}}$, the average library-normalized expression of gene $G_{j}$ within each cluster $T_{k}$, and dividing by $\left\|\mu_{\cdot G_{j}}\right\|$ the sum of the average expression vector across all clusters ($L_{1}$ norm)

$${\hat{\mu }}_{T_{k}G_{j}}\;=\frac{\mu_{T_{k}G_{j}}}{\left\|\mu_{\cdot G_{j}}\right\|}$$

*Cell type specificity of genes.* Relationships between gene and cluster identity were assessed probabilistically. Recall from above that N is the count matrix whose elements $N_{kj}$ are the number of transcripts observed in cluster $T_{k}$ and gene $G_{j}$ and that κ is the total number of transcripts. The probability of observing a specific combination of cluster $T_{k}$ and gene $G_{j}$ is the joint categorical distribution

$$P\left(T_{k},G_{j}\right)=\;\frac{N_{kj}}{\kappa }$$

such that

$$\sum_{k=1}^{n_{\text{clust}}}\sum_{j=1}^{n_{\text{gene}}}P\left(T_{k},G_{j}\right)=1$$

The two marginal distributions are $P\left(G_{j}\right)=\;\left(\left|G_{j}\right|/\kappa \right)$, representing the probability of observing gene $G_{j}$, and $P\left(T_{k}\right)=\left(\left|T_{k}\right|/\kappa \right)$, the probability of observing cluster $T_{k}$. The entropy for a given clustering T is

$$H\left(T\right)=-\sum_{k=1}^{n_{\text{clust}}}P\left(T_{k}\right)\log_{2}\;P\left(T_{k}\right)$$

The gene entropy is given by

$$H\left(G\right)=-\sum_{j=1}^{n_{\text{gene}}}P\left(G_{j}\right)\log_{2}\;P\left(G_{j}\right)$$

The entropy for the joint distribution is

$$H\left(G_{j},T_{k}\right)=-\sum_{k=1}^{n_{\text{clust}}}\sum_{j=1}^{n_{\text{gene}}}P\left(T_{k},G_{j}\right)\log_{2}\;P\left(T_{k},G_{j}\right)$$

We utilize these entropies to assess the cluster specificity of a gene – whether transcripts of the gene are concentrated within some clusters and not others. An entropy of zero means there is no uncertainty (i.e., 100% probability of the gene and cluster identities coinciding) and entropy is at its maximum for perfectly random probability distributions (i.e., equal probability of all gene and cluster combinations). For a transcript from a given gene $G_{j}$, the uncertainty about the cluster identity is the conditional entropy

$$H\left(T|G_{j}\right)=-\sum_{k=1}^{n_{\text{clust}}}P\left(T_{k},G_{j}\right)\log_{2}\;P\left(T_{k},G_{j}\right)$$

The specificity of individual disease-associated genes ${\text{spg}}_{G_{j}}$ was defined relative to the normalized entropy

$${\text{spg}}_{G_{j}}=1-\frac{H\left(T|G_{j}\right)}{\log_{2}n_{\text{clust}}}$$

The significance of ${\text{spg}}_{{\text{G}}_{\text{j}}}$ was assessed using a Monte Carlo permutation test. The following process was repeated a total of $n_{\text{iter}}=1,000$ times: (i) C, the cell identity of transcripts, was randomly shuffled to create C’; meanwhile, its paired observation G was held constant; (ii) the existing Infomap clustering was applied to C’ to generate T’, the cluster identity of the cell-shuffled transcripts; thus, the cell type identities are randomized at the transcript level without changing $P\left(G_{j}\right)$ or $P\left(T_{k}\right)$; (iii) the count matrix with shuffled cell-type identities $N_{kj}^{'}$ was generated from T’ and G; (iv) the conditional entropy of gene $G_{j}$ was determined for the permuted clustering $H\left(T'|G_{j}\right)$ and the specificity ${\text{spg}}_{G_{j}}^{'}$ calculated; (v) the value of ${\text{spg}}_{G_{j}}^{'}$ was stored and if it was greater than the specificity in the non-permuted clustering ${\text{spg}}_{G_{j}}$ a counter b was incremented.

Once concluded, if b>10 then $P\;=\left(\left(1+b\right)/n_{\text{iter}}\right)$, otherwise the parameters φ of a generalized Pareto distribution $F_{\varphi }\left(z\right)$ were fit to the exceedances, z, which are the permutation test occurrences of ${\text{spg}}_{G_{j}}^{'}$ that exceeded the threshold ϕ (Knijnenburg et al., 2009). The initial value of ϕ was set such that there were $n_{\text{exceed}}=250$ exceedances, and the goodness-of-fit for the generalized Pareto distribution was evaluated by the Kolmogorov-Smirnov test. If the fit was poor (null hypothesis that distributions are identical rejected, p < 0.05), ϕ was adjusted upward such that $n_{\text{exceed}}$ was lowered in steps of 10 until the fit was good. The test statistic ${\text{spg}}_{G_{j}}$ was then evaluated on the fit distribution to generate the P value

$$P_{\text{gpd}}=\frac{n_{\text{exceed}}}{n_{\text{iter}}}\left(1-F_{\varphi }\left({\text{spg}}_{G_{j}}-\phi \right)\right)$$

It was also useful to assess the comodulation of a subset of genes, $G^{'}$ across the set of clusters. This method was used to quantify the relationship between gene identity and cluster identity for (i) a set of genes marking different cell types (marker-defined cell types) and (ii) the subset of genes associated with a disease. To do so, we calculated the mutual information between the cluster identities, T, and a subset of gene identities, G'

I(T,G')=H(T)+H(G')−H(T,G')

If the mutual information is not zero, then knowledge of a transcript’s genetic identity reduces the uncertainty about its cluster identity. The reverse is also true, but we focus on the case where genes are given. The cluster identity information gained by knowing the gene identity expressed relative to the uncertainty about cluster identity is the uncertainty coefficient

$$U\left(T|G'\right)=\frac{I\left(T,G'\right)}{H\left(T\right)}$$

the *P*-value of which was estimated by the Monte Carlo permutation test described above with one modification: in steps (iv) and (v) the statistic being tested was the uncertainty coefficient rather than the specificity.

##### Disease genes

The list of disease genes was drawn from the NIH Genetics Home Reference (https://ghr.nlm.nih.gov). We included all genes they listed as being associated with a disease as of August 13^th^ 2018. To ensure an unbiased representation, the list was not revised based on our own observations. In the disease maps, four disease genes were not visualized because they were not expressed in at least one dataset (age-related macular degeneration-associated genes: *F13B*, *CFHR2*, *CFHR3*, and *CFHR5*).

## Supplemental Information

Table S1. Properties of iPSC Lines Tested for Retinal Organoid Formation, Related to Figures 1 and S1

Table S2. Atlas of Cell-Type Gene Expression For Human Peripheral Retina, Foveal Retina, and Developed F49B7 and IMR90.4 Retinal Organoids, Related to Figures 5, 6, 7, S3, S4, S5, and S7 Atlases organized into sheets. Rows, genes. Columns, cell types. Values, expression normalized to 10,000 transcript counts per cell type.

Table S3. The Regional Transcriptomic Character of Adult Peripheral Retina, Adult Foveal Retina, and Developed Organoid Cell Types, Related to Figure S4 Dashed lines, cell type not observed in these developed organoids. Data from F49B7 organoids.

Table S4. Cell-Type Specificity of Retinal Disease-Associated Genes, Related to Figures 7A, 7B, and S7 Organoids recapitulated 69% of the cell-class specificity of disease gene expression. Data from F49B7 organoids.
