# Supplementary material for: Cell Types of the Human Retina and Its Organoids at Single-Cell Resolution
Source: Cell. 2020 Sep 17;182(6):1623–1640.e34. doi: 10.1016/j.cell.2020.08.013 (PMC7505495; doi:10.1016/j.cell.2020.08.013)
Supplement: Table S1. Properties of iPSC Lines Tested for Retinal Organoid Formation, Related to Figures 1 and S1 [file mmc1.pdf]

Cell, Volume *182*

Supplemental Information

Cell Types of the Human Retina and Its Organoids at Single-Cell Resolution

Cameron S. Cowan, Magdalena Renner, Martina De Gennaro, Brigitte Gross-Scherf, David Goldblum, Yanyan Hou, Martin Munz, Tiago M. Rodrigues, Jacek Krol, Tamas Szikra, Rachel Cuttat, Annick Waldt, Panagiotis Papasaikas, Roland Diggelmann, Claudia P. Patino-Alvarez, Patricia Galliker, Stefan E. Spirig, Dinko Pavlinic, Nadine Gerber-Hollbach, Sven Schuierer, Aldin Srdanovic, Marton Balogh, Riccardo Panero, Akos Kusnyerik, Arnold Szabo, Michael B. Stadler, Selim Orgül, Simone Picelli, Pascal W. Hasler, Andreas Hierlemann, Hendrik P.N. Scholl, Guglielmo Roma, Florian Nigsch, and Botond Roska

	Normal control (N) or disease (D)	Cell line	Formation of retinal structues on Matrigel	Maintenance of layered appearance for >100 days	Number of differentiation experiments
1	N	HPS0076: 409B2, Riken Cell Bank	no	no	1
2	N	iPS(IMR90)-1-DL-01, WiCell	no	no	1
3	N	iPS(IMR90)-4-DL-01, WiCell	yes	yes	>30
4	N	ND41866*C, Coriell	no	no	4
5	N	GM23396*D, Coriell	no	no	5
6	N	GM23450*B, Coriell	no	no	4
7	N	01F49i-N-B7	yes	yes	>70
8	N	Internal line 1	yes	yes	>11
9	D	Internal line 2	no	no	1
10	N	Internal line 3	no	no	1
11	N	Internal line 4	no	no	1
12	D	Internal line 5	no	no	1
13	D	Internal line 6	no	no	1
14	N	Internal line 7	no	no	2
15	D	Internal line 8	no	no	1
16	D	Internal line 9	yes	yes	2
17	D	Internal line 10	yes	no	1
18	D	Internal line 11	yes	yes	1
19	N	Internal line 12	yes	yes	2
20	N	Internal line 13	no	no	1
21	D	Internal line 14	yes	no	1
22	N	Internal line 15	yes	yes	1
23	D	Internal line 16	yes	yes	1

Table S1. Properties of iPSC lines tested for retinal organoid formation. Refers to Figure 1, Figure S1.
