# Supplementary material for: Cell Types of the Human Retina and Its Organoids at Single-Cell Resolution
Source: Cell. 2020 Sep 17;182(6):1623–1640.e34. doi: 10.1016/j.cell.2020.08.013 (PMC7505495; doi:10.1016/j.cell.2020.08.013)
Supplement: Table S3. The Regional Transcriptomic Character of Adult Peripheral Retina, Adult Foveal Retina, and Developed Organoid Cell Types, Related to Figure S4 — Dashed lines, cell type not observed in these developed organoids. Data from F49B7 organoids. [file mmc3.pdf]

Cell type or group of cell types	Adult retinal cells	Developed retinal organoid cells	
	Classifier performance, coefficient of determination (R^2)	Peripheral overlapping (% cells)	Foveal overlapping (% cells)
Rods	0.54	63%	8%
Cones	0.89	21%	43%
Horizontal cells	0.69	37%	26%
ON bipolar cells	0.47	25%	13%
OFF bipolar cells	0.44	10%	13%
Amacrine cells	0.46	94%	0%
Ganglion cells	0.66	-	-
Müller cell	0.91	60%	4%
Astrocytes	0.65	-	-
Pigment epithelial cells	0.93	70%	0%
Choroidal melanocytes	0.56	-	-
Microglia	0.48	-	-
Pericytes	0.29	-	-
Fibroblasts	0.90	-	-
Vascular endothelia	0.52	-	-

Table S3. The regional transcriptomic character of adult peripheral retina, adult foveal retina, and developed organoid cell types. Dashed lines, cell type not observed in these developed organoids. Data from F49B7 organoids. Refers to Figure S4.
