# Supplementary material for: Cell Types of the Human Retina and Its Organoids at Single-Cell Resolution
Source: Cell. 2020 Sep 17;182(6):1623–1640.e34. doi: 10.1016/j.cell.2020.08.013 (PMC7505495; doi:10.1016/j.cell.2020.08.013)
Supplement: Table S4. Cell-Type Specificity of Retinal Disease-Associated Genes, Related to Figures 7A, 7B, and S7 — Organoids recapitulated 69% of the cell-class specificity of disease gene expression. Data from F49B7 organoids. [file mmc4.pdf]

Disease	Cell type specificity						
	Peripheral retina		Foveal retina		Developed retinal organoid		
	**Significance (*P*-value)**	**Individual genes significant at *P* < 0.01**	**Significance (*P*-value)**	**Individual genes significant at *P* < 0.01**	**Significance (*P*-value)**	**Individual genes significant at *P* < 0.01**	**Individual genes, same cell class with peak expression as peripheral retina**
Achromatopsia	2×10^{-10}	100%	2×10^{-9}	100%	1×10^{-7}	100%	100%
Congenital stationary night blindness	5×10^{-11}	100%	2×10^{-11}	100%	2×10^{-9}	100%	100%
Retinitis pigmentosa	9×10^{-13}	73%	1×10^{-13}	77%	2×10^{-11}	68%	74%
Leber congenital amaurosis	3×10^{-12}	69%	2×10^{-12}	88%	5×10^{-9}	69%	69%
Macular degeneration	2×10^{-13}	90%	2×10^{-13}	80%	6×10^{-11}	53%	53%
Myopia	6×10^{-11}	57%	3×10^{-11}	43%	3×10^{-9}	57%	29%
Cone-rod dystrophy	3×10^{-10}	97%	4×10^{-11}	84%	2×10^{-9}	90%	77%
Choroideremia	1.0	100%	7×10^{-4}	100%	1.0	0%	100%
Macular dystrophy	1×10^{-10}	100%	1×10^{-10}	100%	4×10^{-9}	100%	100%
Glaucoma	3×10^{-9}	67%	3×10^{-8}	67%	1×10^{-5}	33%	0%
Usher syndrome	2×10^{-8}	91%	2×10^{-8}	100%	6×10^{-7}	73%	82%
Bardet-Biedl syndrome	1×10^{-5}	57%	5×10^{-6}	43%	2×10^{-4}	21%	64%

Table S4. Cell type specificity of retinal-disease-associated genes. Organoids recapitulated 69% of the cell-class specificity of disease gene expression. Data from F49B7 organoids. Refers to Figure 7A - B, Figure S7.
